# OPEX: Development of a novel overall patient experience measure to facilitate interpretation of comparison effectiveness studies

**DOI:** 10.1371/journal.pone.0245598

**Published:** 2021-01-29

**Authors:** Liana Fraenkel, Zhenglin Wei, Christine Ramsey, Carole Wiedmeyer, Kaleb Michaud, Tuhina Neogi, W. Benjamin Nowell, Shilpa Venkatachalam, David A. Broniatowski

**Affiliations:** 1 Department of Medicine, Section of Rheumatology, Yale University School of Medicine, New Haven, Connecticut, United States of America; 2 Department of Medicine, Section of Rheumatology, Berkshire Health Systems, Pittsfield, Massachusetts, United States of America; 3 Department of Engineering Management and Systems Engineering, The George Washington University, Washington, DC, United States of America; 4 Mental Illness Research Education and Clinical Center, Corporal Michael J. Crescenz VA Medical Center, Philadelphia, PA, United States of America; 5 Yale Center for Medical Informatics, Yale School of Medicine, New Haven, CT, United States of America; 6 Patient-Centered Research, Global Heathy Living Foundation, Upper Nyack, NY, United States of America; 7 Division of Rheumatology and Immunology, University of Nebraska Medical Center, Omaha, NE, United States of America; 8 Forward, The National Databank for Rheumatic Diseases, Wichita, KS, United States of America; 9 Department of Medicine, Section of Rheumatology, Boston University School of Medicine, Boston, MA, United States of America; Texas A&M University, UNITED STATES

## Abstract

**Objectives:**

A measure that encompasses both benefits and harms at the individual patient level may facilitate comparisons between treatment options and improve shared decision-making. The objective of this study was to develop a patient reported measure to capture overall experience (including both benefits and harms) of treatment using rheumatoid arthritis (RA) as a case example.

**Methods:**

Hierarchies for treatment benefits are known. Therefore, we developed a hierarchy of adverse events (AEs) using a series of trajectory mapping and paired comparison surveys. We subsequently used these data to construct a paired comparison survey, asking patients to compare options including both a specified level of benefit and an AE. These data were used to generate a hierarchy of overall experience on treatment.

**Results:**

782 participants completed a series of three surveys. The trajectory mapping procedure and a paired comparison survey led to the generation of a hierarchy of AEs with nine levels ranging from No AEs to irreversible serious complications. In a third survey, in which AEs were paired with benefits, participants’ ratings generated a 6-level hierarchy of overall experiences ranging from Major improvement + No, mild or manageable AEs (Level 1) to No improvement + Irreversible AEs (Level 6).

**Conclusions:**

Using a trajectory mapping approach, we developed a patient reported measure representing the distribution of patients’ overall experiences on treatment. The intent of this measure is to enable patients and their physicians to compare the percentage of patients experiencing each level of outcome, from most to least desirable, across treatments.

## Introduction

Comparative effectiveness data allow decision-makers to compare benefits and harms across treatment options. However, studies may not provide patients with all the information they need to make informed choices. Specifically, the results of randomized controlled trials (RCTs) are usually reported as either average improvement scores or the percentage of patients attaining a defined response in each treatment arm. The numbers of participants experiencing specific adverse events (AEs) are reported separately from the numbers of patients reporting improvement. This approach does not quantify what is most important to patients, i.e., their overall experience on treatment. In the words of a patient: “Patients have no way to determine the potential net benefit for a given treatment, much less to compare across treatments. How will I feel overall on medication A compared to medication B?” Simultaneously weighing the efficacy and AEs of multiple drugs is also challenging for physicians and makes it difficult for them to effectively engage their patients in shared decision-making. Thus, there is a need for more informative evaluation measures.

Some measures encompassing both benefits and risks have been developed. Net clinical benefit subtracts a predefined negative outcome from a predefined benefit in treatments with narrow therapeutic windows, such as warfarin to prevent stroke in patients with non-valvular atrial fibrillation [1]. Quality-adjusted time without symptoms of disease progression or toxicity of treatment (Q-TWiST) combines utilities associated with three health states in cancer trials: toxicity, time without symptoms and toxicity, and relapse [2]. Desirability of outcome ranking (DOOR) generates a ranking of patients based on both clinical outcomes and duration of antibiotic use [3]. To date, however, measures reporting the percentage of patients with varying overall experiences have yet to be developed.

A rank ordering of patients’ overall experience (AEs and improvements) on medications must be constructed based on patients’ values. Some preference-based methods, such as conjoint analysis, assign a raw number to each characteristic with the aim of establishing a “total order” over the full set of characteristics [4]. Thus, for any given pair of characteristics, one can always identify a preferred characteristic. This approach obfuscates the fact that there are some ways in which one characteristic may be better, and other ways in which it may be worse, than others. For example, when comparing AEs of medications used to treat rheumatoid arthritis (RA), there may be some ways in which patients feel that cataracts are better than pneumonia, and other ways in which they are worse. In addition, some treatment characteristics or outcomes may be perceived by patients as too different to compare. Thus, patients may only be able to partially order alternatives, i.e. they may only be able to indicate whether one is “better”, “worse”, “the same”, or “incomparable” to another.

We therefore based our approach on Trajectory Mapping (TM) [5]. TM is a non-metric scaling technique that may be used to organize and extract relationships between stimuli (such as risk/benefit profiles). Unlike metric scaling approaches that impose total ordering, such as multidimensional scaling and hierarchical clustering, TM assumes that objects are represented as discrete categories that can, but need not, be ranked relative to one another. This partially ranked approach allows the construction of equivalence classes, i.e., groups of outcomes that may not be directly comparable, but can all be compared relative to a common reference point. For example, TM enables people to state that two objects are either better than (or worse than) another reference object, even though they may not be directly compared with one another. Thus, it allows patients to say, for example, that while pneumonia and cataracts may not be directly comparable, they are both worse than heartburn.

The objective of this study was to develop a patient reported measure to capture overall experience on treatment, which we refer to as the **O**verall **P**atient **EX**perience measure (OPEX). In this context, we define overall experience as encompassing both the level of benefit and AEs experienced by an individual patient. Our approach was informed by Evans et al.’s DOOR [3].

We conducted two sequential studies using RA as a case example. AE lists with high content validity have been developed for cancer patients (e.g., PRO-CTCAE; [6]). Furthermore, studies have shown that patients are able to distinguish between AEs on the basis of severity in pairwise comparisons [7]. However, these lists were not designed for RA patients; nor do they rank order AEs in a partially ordered hierarchy. Our first study was designed to generate a hierarchy of AEs for RA patients. Given this hierarchy of AEs, we conducted a second study to combine AEs with benefits, enabling us to generate a hierarchy of overall experiences on RA treatments ranked from most to least desirable.

## Methods

This study was deemed IRB exempt under Schulman (now Advarra) IRB protocol # 201705022. For all surveys, subjects indicated consent by clicking an invitation to continue to the survey.

### Study 1: Building an AE hierarchy

#### Overview of study procedures

The aim of this study was to develop an AE hierarchy that can be used to directly compare AEs to each other whenever possible. We recognize that some AEs may not be directly comparable, thus our approach was to construct a partial ordering, allowing us to segment AEs into “equivalence classes”–i.e., groups that could be ranked relative to one another, even if the AEs themselves might not be directly comparable within groups. We recruited RA patients to complete a TM survey to order AEs relative to one another as “better”, “worse”, or “incomparable”. We then constructed several candidate AE hierarchies using the TM data and employed a model selection procedure to remove hierarchies that did not fit the data well. Lastly, we fielded a survey to evaluate the predictive accuracy of the remaining hierarchies and to select the final best-fitting AE hierarchy. Each step is described in greater detail below and in the S1 Appendix. The study was approved by the participating institutions’ human research protection programs.

#### Selection and description of participants

Participants were recruited by the Global Healthy Living Foundation, through the organization’s CreakyJoints (CJ) and ArthritisPower online patient communities, and by advertisements displayed to fans of the CJ Facebook page. CJ is a large arthritis patient network of more than 100,000 patients. At the time of this study, 84% of CJ members were female, 82% were white, the average age was 54 (SD 12), and 52% self-reported having RA. ArthritisPower is a Patient-Centered Outcomes Research Institute (PCORI)-funded patient-designed research registry of approximately 18,000 people with joint, bone, and inflammatory skin conditions, with about 46% self-identifying as having RA. At the time of this study, 91% were female, 88% were White, and the average age was 53 (SD 11; see S1 Appendix for more demographic information). Eligibility criteria included English speaking adults, reporting a diagnosis of RA made by a physician, and currently taking a disease modifying antirheumatic drug (DMARD). For the initial survey eliciting descriptions of AEs, participants were also eligible if they had taken a DMARD in the past but were not currently taking one.

Participants were recruited via email invitations. Screening questions were included in the survey to confirm eligibility criteria. Invitations included unique links that allowed respondents to take the survey one time. Since an individual could use multiple email addresses to receive more than one link, we de-duplicated by name and address before sending the invitations. We also checked the data to see if someone took the survey twice. Three duplicate responses were removed. Participants who completed a survey were excluded from receiving an invitation to participate in subsequent surveys.

#### Eliciting AEs

We performed an online survey to elicit patient descriptions of AEs they experienced while on glucocorticoids and/or disease modifying antirheumatic drugs (DMARDs) used to treat RA. For each AE, patients were asked to describe the frequency and severity of the AE, whether any treatment was necessary, the impact of the AE on the patient’s quality of life, and the outcome. We developed a list of AEs based on the content generated by patients’ descriptions. These descriptions were subsequently presented to a patient panel during a webinar for their feedback. The revised list of AEs (Table 1) was used in the subsequent TM and paired comparison surveys. Patients were provided with a detailed description of each AE including the characteristics they stated were needed to be able to rate each AE: frequency, severity, management, and reversibility.

**Table 1:** Adverse event descriptions.

Label	Adverse Event	Adverse Event Description
A	Manageable headaches	Infrequent and/or tolerable severity, manageable with over-the-counter medications such as Tylenol, diet or lifestyle changes. Frequency varies, but usually less than once a week.
B	Severe headaches	Severe or frequent enough to affect quality of life and/or limit activities. Frequency varies, but on average happens 4 times per month.
C	Manageable fatigue and/or insomnia	Infrequent and/or tolerable severity, manageable with over-the-counter medications, diet or lifestyle changes. Frequency varies, but usually less than once a week. Usually happens after a particularly active day or a lot of stress.
D	Severe fatigue and/or insomnia	Severe or frequent enough to affect quality of life and/or limit activities. Frequency varies, but usually happens at least 2 to 3 days a week—regardless of the amount of activity or stress going on.
E	Manageable brain fog and/or lightheadedness	Infrequent and/or tolerable severity, manageable with over-the-counter medications or lifestyle changes. Frequency varies, but usually less than once a week—and manageable with rest.
F	Severe brain fog and/or lightheadedness	Severe or frequent enough to affect quality of life and/or limit activities. Frequency varies, but usually happens at least 2 to 3 days a week.
G	Manageable depression and/or anxiety	Infrequent and/or tolerable severity, manageable with over-the-counter medications, lifestyle changes, or mind-body therapies. Frequency varies but may have 2 to 3 episodes lasting a couple of weeks a year.
H	Cataracts	Requires surgery to fix. Need to take it easy for 2 to 3 days after the surgery.
I	Change in appearance (hair thinning or loss, or weight gain or loss)	Weight gain is usually in the stomach area, but some patients can get a moon face. Reversed when RA medication dose is lowered or stopped.
J	Manageable mouth ulcers	Infrequent and/or tolerable severity, manageable with over-the-counter medications. Frequency varies, but usually happens less than once a month and are more of a nuisance than painful.
K	Manageable stomach pain and/or diarrhea	Infrequent and/or tolerable severity, manageable with over-the-counter medications, or change in diet. Frequency varies, but usually less than once a week. Tends to get better with time.
L	Manageable nausea and/or vomiting	Infrequent and/or tolerable severity, manageable with over-the-counter medications or change in diet. Frequency varies, but usually less than once a week. Tends to get better with time.
M	Mild injection site skin reaction or rash	Mild and tends to resolve with continued use of RA medication.
N	Mild infection treated at home	Treated with oral antibiotics at home; includes: bronchitis, head cold (sinusitis) or bladder infection, etc. Frequency varies but can happen anywhere from 2 to 3 times per year.
O	Shingles	Treated with oral medication at home. Shingles very rarely happens more than once. But about 20% of patients can continue to have pain where the rash was, even after the rash is completely healed.
P	Serious infection treated in the hospital	Requires intravenous (IV) medications and about 5 days in the hospital; takes 2 to 3 weeks to fully recover. Includes: pneumonia, skin infections, urinary tract infections, etc. Frequency varies but can happen once per year.
Q	Curable non-melanoma skin cancer	Cured with topical medication or surgery. Recovery depends on the lesion(s), but is usually easy with a bandage over the affected skin.
R	Reversible blood test abnormalities	Does not cause any symptoms. Affects liver, kidney or blood cells. Reversed when RA medication dose is lowered or stopped.
S	Diet-controlled high blood pressure, sugar, or cholesterol	Ongoing condition. Manageable with change in diet and/or exercise. May reverse when RA medication dose is lowered or stopped.
T	Medication-controlled high blood pressure, sugar, or cholesterol	Ongoing condition. Requires adding blood pressure, blood sugar or cholesterol medication. May reverse when RA medication dose is lowered or stopped.

#### Ordering AEs using trajectory mapping

We subsequently conducted a TM survey to generate a set of candidate AE hierarchies. The TM task included the following consecutive steps (see the “Trajectory Mapping” section in the S1 Appendix). Participants were presented with a randomly selected pair of AEs and asked to indicate which is worse. For example, when comparing severe headaches (Severe or frequent enough to affect quality of life and/or limit activities. Frequency varies, but on average happens 4 times per month.) (B) to a mild infection treated at home (Treated with oral antibiotics at home; includes: bronchitis, head cold (sinusitis) or bladder infection, etc. Frequency varies but can happen anywhere from 2 to 3 times per year.) (N), a participant might indicate that the former is worse. Next, participants identified the specific criterion on which they made their choice. For example, a participant might indicate that severe headaches interfere more with daily activities than do mild infections (B > N). Participants then extrapolated outcomes using the same criterion. Continuing the preceding example, participants would then be asked to select an AE that interferes less with daily activities than a mild infection [e.g., mild injection site skin reaction or rash (M)] and an AE which interferes more with daily activities than severe headaches [e.g., serious infection treated in the hospital (P)]. Finally, participants selected an AE that is intermediate between the two original AEs [e.g., Manageable nausea and/or vomiting (L)]. The result was an ordered quintuple of AEs (M > N > L > B > P, where “X > Y” indicates that X is preferred to Y), which was then split into three triples (M > N > L, N > L > B, L > B > P). Each participant completed the TM task for 11 pairs of AEs. We tested the hypothesis that each triple occurred more frequently than would be expected if AEs were chosen uniformly at random without replacement. After controlling for multiple comparisons using the Holm-Bonferroni procedure, all triples that appeared more frequently than would be expected under this null hypothesis were retained (further details are provided in the S1 Appendix).

#### Constructing a Hierarchy of AEs

All edges in the retained triples were included in an adjacency matrix to construct a network representing all ordered relations between AEs. To summarize these data, we used five different network analysis algorithms (see S1 Appendix) to generate several potential hierarchies from this adjacency matrix [7–10]. For each hierarchy, we evaluated how well it fit the data using standard model goodness-of-fit measures (the Akaike and Bayesian Information Criteria; AIC and BIC) based on a logistic loss function shown in Eq 1, where *n*_*A*_ is the number of triples expressing a preference for option A over option B, and k is chosen to minimize the value of ℒ (see S1 Appendix), with the goal of retaining the best-fitting hierarchy.

$$L=-\sum_{A,B}\left[n_{A}*\text{ln}\left(P\left(A,B\right)\right)+n_{B}*\text{ln}\left(P\left(B,A\right)\right)\right]+k^{2}$$ (1)

### Study 2: Building the OPEX

#### Overview of study procedures

In the second study we used a paired comparison survey to generate a hierarchy of overall experiences describing a level of benefit and an AE (OPEX).

#### Constructing levels of benefit

Three benefit levels were chosen based on patient input (KM, CW) and to correspond as much as possible to the American College of Rheumatology response criteria [8] (the ACR 20, 50 and 70):

- Major improvement–Most or all joint pain, swelling, and stiffness resolved
- Some improvement–Some joint pain, swelling, and stiffness resolved
- Little or no improvement–Very little or no joint pain, swelling, and stiffness resolved

In the instructions presented prior to completing the survey, we explained that “major improvement” equated to between 50% and 100%, “some improvement” to between 20% to 49%, and “little or no improvement” to less than 20% improvement in joint pain, swelling and stiffness.

#### Overall experience hierarchy survey

We asked participants to rate their preference for pairs of overall experiences containing a specified level of benefit and an AE. Pairs in which one option had a greater benefit and milder AE compared to the other were not included. Further details regarding the design of the survey are included in the S1 Appendix. Using pilot data from the prior surveys, we conducted a power analysis, which indicated that 401 subjects evaluating 25 AE/benefit profiles each were required to detect a difference of at least 0.8 Likert-scale points between levels of the overall experience hierarchy (the minimum difference between items on different levels in prior surveys) with a Type I error rate of alpha = 0.05 after controlling for multiple comparisons using the Bonferroni procedure.

## Results

### Study 1

#### Eliciting AE descriptions from patients

Forty-six participants [mean (SD) age = 54 (11), 91% female, 96% White] (see S1 Appendix for more demographic information) provided descriptions of AEs which were used to create a set of 20 AEs (Table 1). Given these 20 AEs, a power analysis for the TM survey indicated that 173 subjects, completing 11 quintuples each, were needed to achieve statistical significance (see S1 Appendix).

#### Ordering AEs using trajectory mapping

The TM survey was completed by 200 patients with RA. We retained data from 195 participants (97.5%) after eliminating five subjects who gave the same answer to all questions or who finished in less than 20 minutes. The mean (SD) survey duration was 49 (16) minutes. Most respondents were female (89%) and white (93%). Their mean (SD) age was 52 (11), 92% had a college degree or higher, and 48% rated their overall health as fair or poor (see S1 Appendix for more demographic information).

The TM survey responses generated 1,897 quintuples yielding a total of 5,195 ordered triples. Eighty-three of these ordered triples occurred six times or more and were therefore statistically significant at the p<0.05 level after adjusting for multiple comparisons (see S1 Appendix). The criteria used most frequently by participants to compare AEs were “How much it affects overall quality of life” (21% of total comparisons; 85% of subjects used this criterion at least once) and “How much it affects ability to do activities” (12% of total comparisons, 63% of subjects used this criterion at least once).

The initial analysis of TM data generated 11 hierarchies of AEs. We were unable to select a single best-fitting hierarchy based on AIC and BIC values alone. The four best -fitting hierarchies were selected for further evaluation (Table 2). These four hierarchies displayed a similar preference structure. For example, all four found that “mild injection skin reaction or rash” is the most preferred and that “serious infection treated in the hospital” is the least preferred AE.

**Table 2:** Candidate adverse event hierarchies.

	Candidate Hierarchy			
Level	H1	H2	H3	H4
1	A	A	A	A
2	B, C	B	B, C, E	B, C
3	D, E, F	C, D, E	D, F, G	D, E, F
4	G, H	F, G	H	G
5	I	H	I	H
6	J	I	J	I
7		J		J

A: Mild injection site reaction or rash.

B: Manageable headaches, Manageable mouth ulcers, Mild infection treated at home, Reversible blood test abnormalities.

C: Manageable fatigue and/or insomnia, Manageable brain fog and/or lightheadedness, Diet-controlled high blood pressure, sugar, or cholesterol.

D: Manageable depression and/or anxiety, Manageable stomach pain and/or diarrhea, Manageable nausea and/or vomiting.

E: Change in appearance (hair thinning or loss, or weight gain or loss).

F: Medication-controlled high blood pressure, sugar, or cholesterol.

G: Cataracts, Curable non-melanoma skin cancer.

H: Severe fatigue and/or insomnia, Severe brain fog and/or lightheadedness.

I: Severe headaches, Shingles.

J: Serious infection treated in the hospital.

We identified nine groups of AEs (represented by unique colors in Table 2) which consistently appeared together, indicating that participants were either indifferent or could not indicate a preference between the AEs within each of these groups. The overall ranking of groups of AEs was similar across the four hierarchies. Some groups consistently fell into the same level of the hierarchy. For example, manageable headaches, manageable mouth ulcers, mild infections treated at home, and reversible blood test abnormalities were viewed as being on Level 2 in all four hierarchies. However, other groups fell into different levels. For example, cataracts and curable non-melanoma skin cancer fell into Level 4 for all but one hierarchy, and manageable fatigue, brain fog/lightheadedness, diet controlled high blood pressure, sugar, or cholesterol fell into Level 2 for three of the four hierarchies. Groups of AEs differed between hierarchies by only a single level, except for in the case of severe headaches and shingles, which fell into Level 4 in some hierarchies and Level 6 in others.

#### AE hierarchy validation survey

To resolve differences between the four best-fitting hierarchies, we conducted a second paired comparison survey. The hierarchies differed on the relative locations of 11 pairs of AE groups. Thus, in the validation survey, participants were asked to indicate which group of AEs, within each of these 11 pairs, was worse, using a 7-point Likert scale (see S1 Appendix). Based on pairwise comparison data derived from the TM survey, a power analysis for the AE validation hierarchy indicated that each pair needed to be evaluated 90 times to detect a difference in preference between AEs of least one Likert-scale point after controlling for multiple comparisons using the Bonferroni procedure. Email invitations were sent to 3,132 ArthritisPower members and 129 completed the survey. The mean (SD) survey duration was 6 (2) minutes. We discarded the data from two subjects who completed the survey in under two minutes, leaving responses from 127 (98%) participants [89% female, mean (SD) age = 55 (11)] (see S1 Appendix for more demographic information).

Of the four candidate AE hierarchies, a 6-level AE hierarchy yielded the best AIC and BIC values. This result was replicated using several approaches (see S1 Appendix). We found that the distribution of subjects’ ratings for one AE, change in appearance (hair thinning or loss, or weight gain or loss), was bimodal, indicating multiple interpretations of this AE (all other pairwise comparison ratings were unimodal). Therefore, we split this AE into two categories with different severities (Mild and Moderate).

In order to ensure that the full spectrum of risks, including those of irreversible serious harm (even when rare), was disclosed to patients, we added three additional AE levels with a priori ranking: No AEs, serious complication from which you fully recover (like a bowel perforation that requires emergency surgery), and serious complication that is irreversible (like a neurologic disease that needs ongoing treatment). Patients may not be familiar with the fatality rates associated with specific outcomes, and might, instead focus on the possible experience of a serious event that one would nevertheless recover from, vs. one that would require ongoing treatment. Thus, these descriptions were provided as examples to communicate the bottom-line risks, such as that the patient would eventually recover, or that the patient might have a chronic illness. To validate these descriptions, these AEs were developed in collaboration with a patient researcher (CW) and then discussed with a patient panel during a webinar. These three levels were not included in the TM or AE hierarchy surveys because their rank orders (best, second worst, and worst, respectively) are known. The final 9-level AE hierarchy is shown in Table 3.

**Table 3:** Final adverse event hierarchy.

Level	Adverse Events													
1	No side effect													
2	Mild injection site skin reaction or rash													
3	Manageable headaches	Manageable mouth ulcers		Mild infection	Reversible blood test abnormalities			Manageable fatigue or insomnia		Manageable brain fog or lightheadedness		Diet-controlled high blood pressure, sugar, or cholesterol		Mild change in appearance
4	Manageable depression or anxiety		Manageable stomach pain or diarrhea			Manageable nausea or vomiting			Medication-controlled high blood pressure, sugar, cholesterol				Moderate change in appearance	
5	Cataracts			Curable non-melanoma skin cancer			Severe fatigue or insomnia				Severe brain fog or lightheadedness			
6	Severe headaches						Shingles							
7	Severe infection treated in the hospital													
8	Serious complication from which you fully recover (like a bowel perforation that requires emergency surgery)													
9	Serious complication that is not fully reversible (like a neurologic disease that needs ongoing treatment)													

### Study 2

#### Overall experience hierarchy survey

Email invitations were sent to 4,895 CJ and ArthritisPower community members of whom 453 completed the survey. The mean (SD) survey duration was 19 (6) minutes. We discarded 27 participants who chose the last option for all questions or who finished the survey in under nine minutes, leaving responses from 426 (94%) participants. Most (91%) were female and white (93%). Their mean (SD) age was 54 (11), 88% had a college degree or higher, and 53% had fair or poor self-rated overall health. We checked for, and did not find, order effects (see S1 Appendix).

Ratings for AEs when paired with benefits show that when evaluated with a specified level of benefit, AEs clustered into four groups: No, mild or manageable AEs (Levels 1–4), Moderate AEs (Levels 5 and 6), Serious AEs (Levels 7 and 8), and Irreversible serious AEs (Level 9). Participants’ ratings (included in the S1 Appendix) generated a 6-level hierarchy of overall experiences (Table 4) ranging from major improvement with no, mild or manageable AEs (Level 1) to little or no improvement with irreversible serious AEs (Level 6). Several equivalence classes emerged. For example, ratings of paired comparisons including one of the three specified levels of benefit and one of the 20 AEs (Table 1) revealed that participants’ valuations of a major improvement associated with moderate AEs were similar to some improvement with no, mild or manageable AEs.

**Table 4:** Global outcome hierarchy.

Level							
1	Benefit	Major improvement					
	AE	No, mild, or manageable AEs					
2	Benefit	Major improvement			Some improvement		
	AE	Moderate AEs			No, mild, or manageable AEs		
3	Benefit	Major improvement		Some improvement		Little or no improvement	
	AE	Serious AEs		Moderate AEs		Mild AEs	
4	Benefit	Major improvement		Some improvement		Little or no improvement	
	AE	Irreversible serious AEs		Serious AEs		Moderate AEs	
5	Benefit	Some improvement			Little or no improvement		
	AE	Irreversible serious AEs			Serious AEs		
6	Benefit	Little or no improvement					
	AE	Irreversible serious AEs					

#### Applying the OPEX to the results of a clinical trial

**Fig 1:**
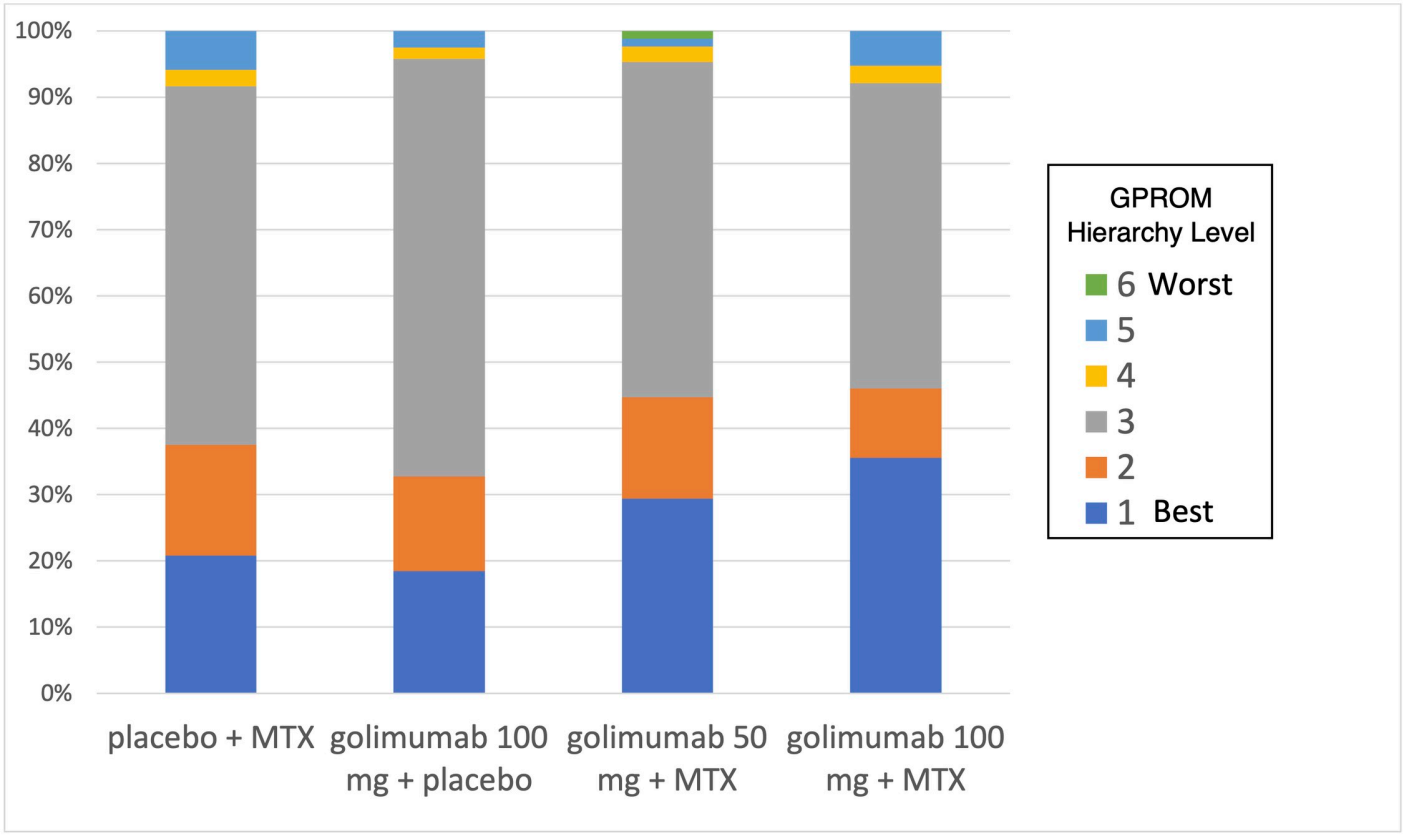
Example presentation of OPEX score distributions.

We obtained individual participant data from the GO-FORWARD study [9], comparing methotrexate, golimumab, and combined therapy options using OPEX. Data from these trials reported all side effects that were part of OPEX, allowing us to map each patient’s outcomes to an OPEX score. In this trial, patients on stable doses of methotrexate were randomized into four regimens: 1) methotrexate + placebo (Group 1; n = 133), 2) 100 mg of golimumab + placebo (Group 2; n = 133), 3) 50 mg of golimumab + methotrexate (Group 3; n = 89), and 4) 100 mg of golimumab + methotrexate (Group 4; n = 89). Benefits were greater in both combination therapy arms (Groups 3 and 4) to monotherapy (Groups 1 and 2). Approximately 30% had very little or no joint pain, swelling, and stiffness at 52 weeks in the monotherapy groups.^15^ This endpoint was reached in 42% in the lower dose combination group (Group 3) and 53% of the higher dose (Group 4) combination group. Any adverse event occurred in 73.7, 81.2, 78.8 and 85.9 percent of the participants in Groups 1 through 4; whereas serious adverse events occurred in 4.5, 12.0, 8.0, and 18.3 percent of participants across the four groups [9]. The distribution of OPEX scores (in which the AEs score was classified based on the most serious AE experienced by each participant) is illustrated in Fig 1. From this figure, patients would be able to see that a greater number of patients on combination therapy fall into the most desired Level (major improvement with no, mild or manageable AEs) compared to those on monotherapy. We also can see that greater number of subjects fall into Level 3 on monotherapy compared to combination therapy. It is also possible to examine more detailed results within each level of OPEX. For example, almost all participants (98%) falling into Level 3 had little or no improvement and mild AEs in all four arms of the trial.

## Discussion

Central to the TM method is the acknowledgement that not all treatment characteristics are directly comparable, but instead may be comparable to a third reference point. The partially-ordered hierarchical representation, extracted in Study 1, enables such comparisons without requiring a direct, and fully transitive, ranking of all characteristics. This approach allowed us to construct a hierarchy of AEs including several equivalence classes of AEs. Furthermore, given a set of AEs, such as those included in the US National Cancer Institute’s PRO-CTCAE, this approach can be applied to other domains outside of RA. This partially-ordered classification is different from the current AE taxonomies, such as the Common Terminology Criteria for Adverse Events, and may be a valuable alternative method of summarizing data when communicating risk. For example, while headaches, insomnia and fatigue are typically classified as non-serious AEs in RCTs, the data from this study demonstrate that reversible non-serious AEs can have an impact ranging from Level 1 through Level 6 on the AE hierarchy (Table 3).

In Study 2, we used a multistep approach to generate OPEX, a patient reported measure able to represent the distribution of patients’ overall experiences on treatment. The data provide a more holistic picture of treatment effects compared to the discrete benefit and harm data currently published. The intent of OPEX is to enable patients and their physicians to compare the percentage of patients experiencing each level of experience, from most to least desirable, across treatments. This tool, once validated, may help patients further distinguish between treatments and support shared decision making. For example, some may prioritize the chance of benefits and choose treatment with a higher percentage of patients in the top Levels (1, 2 or 3). Whereas, others may focus primarily on minimizing risk, and prefer the medication with the fewest patients in Levels 4 through 6.

There is no standard method to compare the combined benefits and AEs experienced by individual patients taking a specified medication. We developed a new measure to enable such comparisons. OPEX classifies patients using scores ranging from 1 to 6, where 1 = Major improvement and no, mild or manageable AEs to 6 = Little or no improvement and irreversible serious AEs. Interestingly, when asked to rate overall experiences, participants treated AEs as falling into one of only four main categories. This approach is consistent with Fuzzy-Trace Theory, which posits that people categorize information into only as much detail as required for them to make a choice or decision [10]. We hypothesize that patients expect some level of toxicity with medications used to treat RA, and think of AEs as either mild and manageable, moderate and having an impact on quality of life, serious i.e., that require burdensome treatment and may be long-lasting, or resulting in irreversible harm. This interpretation was triangulated and endorsed by patients in a follow-up webinar with RA patients.

Central to the trajectory mapping method is the acknowledgement that not all characteristics may be directly comparable but may instead require comparison to a third reference point. The partially-ordered hierarchical representation enables such comparisons without requiring a direct, and fully transitive, ranking of all treatment characteristics. A second novel contribution of this work stems from the hierarchies constructed. Although other investigators have examined pairwise comparisons between specific AEs, ours is the first study, to the best of our knowledge, to construct a holistic partially-ordered hierarchy of AEs, holding benefit levels constant. Our results demonstrate that allowing patients to compare AEs against a third reference point enables construction of equivalence classes of AEs that go beyond simple severity descriptions. Although, severity is correlated with ranking, as expected, severity does not capture the whole of subjects’ AE rankings. For example, manageable fatigue or insomnia is preferable to manageable nausea or vomiting, even though both are “manageable”. The rank ordering of AEs reflected study participants’ views of the impact of AEs on quality of life. This classification is different from current approaches that examine pairwise comparisons between AEs, such as the Common Terminology Criteria for Adverse Events in that it explicitly constructs a *partially-ordered* hierarchy which acknowledges that some AEs cannot be explicitly ranked relative to one another but instead fall into “equivalence classes”. Additionally, our AE hierarchy highlights differences from standard rankings used in RCTs: while headaches, insomnia and fatigue are typically classified as non-serious AEs in RCTs, the data from this study demonstrate the importance of considering the impact of symptoms on patients’ quality of life when comparing specific treatment options.

### Limitations and future work

There are several limitations to this study. Most importantly, while our results demonstrate feasibility of the approach used, these findings are limited to RA. Future work is needed to determine whether we can apply this approach to other populations.

Additionally, in the process of collapsing complex experiences into 6-level hierarchy, some nuances may have been lost. Although over-simplification is always a risk when describing complex patient experiences, our approach retains more nuance than utility-based approaches that seek to collapse these complex experiences into a single numerical rating, or approaches that do not attempt to compare side effects and benefits in a principled way. Additionally, patients were involved at each step of the development and initial testing of the measure, and we triangulated our findings with feedback from patients. Ultimately, OPEX is not intended to replace a detailed discussion with a rheumatologist; rather, it can help provide some clarity and serve as a springboard for discussing what matters to the individual patient.

Patient reports of AEs are limited by difficulties related to attribution. Determining which, if any, medications are causing a specific AE is difficult. OPEX uses the same approach as RCTs, in that ratings are based on outcomes that patient experience regardless of attribution. In addition, we recognize that people are poor forecasters of their responses to future health states. However, all patients must make decisions about treatment without having personally experienced the outcomes they are asked to consider.

In this study, we could not include all possible AEs. Commonly occurring AEs which would be too difficult to tolerate and likely not allow an investigational drug to progress to a Phase III trial (e.g., severe vomiting or depression), were not included. Furthermore, overall experiences were created using a only a single side effect despite the fact that patients might experience several side effects simultaneously. Additional research is needed to determine how best to classify multiple AEs experienced at the individual patient level.

Patients recruited through online arthritis communities do not represent a population-based sample and the study population included few men and minority patients. However, RA is more common in women. The participation rate in each survey was low. This is not unusual given that we were limited to using an opt-in approach and the tasks were challenging and time consuming. However, we were able to recruit sufficient numbers of patients to reach the required sample size for each survey. Future work will need to examine the external validity of OPEX. Lastly, condition eligibility was ascertained based on self-report of physician diagnosis of RA and current use of a DMARD and/or biologic and was not confirmed by medical record or claims data.

Further studies are now required to refine and validate OPEX. For example, future research will enable further comparisons in newer trials, such as the recent RCT comparing a Jak inhibitor to a TNFi–in which the former was found to be more effective but at a higher risk of toxicity [11]. Other notable examples include high risk decisions in which the patient experience is markedly different between two options, such as early rhythm control versus usual care for atrial fibrillation.

We hope that once validated, OPEX will enable RCTs to report the percentage of patients classified into each level; thus, providing patients and their providers with a much clearer understanding of the range and likelihood of the total effects of competing treatment options on their quality of life. If successful, OPEX will improve evidence-based, patient-centered decision making and add value to the outcomes used in comparative effectiveness studies.

## Supporting information

S1 Appendix (PDF)
