# Correction: OPEX: Development of a novel overall patient experience measure to facilitate interpretation of comparison effectiveness studies

**DOI:** 10.1371/journal.pone.0248598

**Published:** 2021-03-09

**Authors:** The PLOS ONE Staff

The Data Availability statement for this paper is incorrect. The correct statement is: All de identified data required to replicate the results in this paper are either already available in the technical appendix or are available at https://github.com/broniatowski/OPEX. Data pertaining to the GO-FORWARD study are available from the Yale University Open Data Access Project at https://yoda.yale.edu/request-data.

The publisher apologizes for the error.

The Acknowledgments section is missing from the paper. The correct Acknowledgments section is: This study, carried out under YODA Project # 2017–2326, used data obtained from the Yale University Open Data Access Project, which has an agreement with JANSSEN RESEARCH & DEVELOPMENT, L.L.C.. The interpretation and reporting of research using this data are solely the responsibility of the authors and does not necessarily represent the official views of the Yale University Open Data Access Project or JANSSEN RESEARCH & DEVELOPMENT, L.L.C.
