# Supplementary material for: OPEX: Development of a novel overall patient experience measure to facilitate interpretation of comparison effectiveness studies
Source: PLoS One. 2021 Jan 29;16(1):e0245598. doi: 10.1371/journal.pone.0245598 (PMC7846019; doi:10.1371/journal.pone.0245598)
Supplement: S1 Appendix — (PDF) [file pone.0245598.s001.pdf]

Technical Appendix

Table S1. Demographics of Survey Respondents

		Study 1				Study 2	
		AE Elicitation Survey		Hierarchy Validation Survey		OPEX Hierarchy Survey	
		N	%	N	%	N	%
Medication	Actemra	15	4%	7	3%	35	4%
	Amjevita (Humira biosimilar)					0	0%
	Arava (Leflunomide)	21	5%	20	9%	44	5%
	Cimzia	7	2%	3	1%	15	2%
	Enbrel	28	7%	15	7%	69	8%
	Erelzi (Enbrel biosimilar)	0	0%	1	0%	0	0%
	Humira	23	6%	19	9%	47	5%
	Inflectra (Remicade biosimilar)					2	0%
	Kevzara	0	0%	1	0%	3	0%
	Methotrexate	100	25%	47	22%	192	22%
	Orencia	27	7%	12	6%	58	7%
	Plaquenil (Hydroxychloroquine)	52	13%	34	16%	135	16%
	Prednisone	50	13%	26	12%	115	13%
	Remicade	16	4%	5	2%	19	2%
	Rituxan	7	2%	2	1%	27	3%
	Simpon	4	1%	4	2%	16	2%
	Sulfasalazine	20	5%	12	6%	39	5%
	Xeljanz	24	6%	10	5%	49	6%
Number of medications used per person	1	70	36%	49	39%	140	33%
	2	75	39%	46	36%	182	43%
	3	38	20%	25	20%	73	17%
	4	10	5%	4	3%	22	5%
	5	1	1%	2	2%	5	1%
	6	1	1%	1	1%	1	0%
	>6	0	0%	0	0%	2	0%
Gender	Male	22	11%	14	11%	37	9%
	Female	173	89%	113	89%	389	91%
Age	20-29	4	2%	3	2%	6	1%
	30-39	22	11%	7	6%	48	11%

	40-49	51	26%	27	21%	94	22%
	50-59	63	32%	46	36%	144	34%
	60-69	42	22%	33	26%	101	24%
	70-79	13	7%	11	9%	30	7%
	80-89	0	0%	0	0%	3	1%
	Mean (SD)	52.38	11.42	54.86	10.89	53.78	11.25
Race	American Indian or Alaska Native	5	3%			13	3%
	Asian	1	1%			2	0%
	Asian Indian	0	0%			1	0%
	Black or African American	3	2%			20	5%
	Other	5	3%			6	1%
	Pacific Islander	0	0%			2	0%
	White	181	93%			395	93%
Hispanic Ethnicity	Yes	13	7%			14	3%
	No	182	93%			412	97%
Education	8th grade or less	1	1%			1	0%
	Some high school, but did not graduate	2	1%			0	0%
	High school graduate or GED	13	7%			49	12%
	Some college or 2-year degree	73	37%			150	35%
	Undergraduate degree	52	27%			117	27%
	Graduate degree	54	28%			109	26%
Occupation	I am a homemaker	18	9%			34	8%
	I am a student	4	2%			5	1%
	I am on disability and do not work	41	20%			127	30%
	I am retired	36	17%			93	22%
	I am unemployed	7	3%			12	3%
	I work full time	71	34%			138	32%
	I work part time	33	16%			48	11%
Health condition	Poor	20	10%			37	9%
	Fair	75	38%			188	44%
	Good	71	36%			145	34%

	Very good	26	13%		48	11%
	Excellent	3	2%		8	2%
Rating over past week (1-Very Well, 11-Very Poor)	1	10	5%		14	3%
	2	13	7%		23	5%
	3	19	10%		36	8%
	4	22	11%		49	12%
	5	11	6%		51	12%
	6	31	16%		65	15%
	7	24	12%		68	16%
	8	25	13%		74	17%
	9	23	12%		32	8%
	10	13	7%		8	2%
	11	4	2%		6	1%
	Mean (SD)	6.04	2.63		4.89	2.28
Household Income	Less than $20,000	18	9%		49	12%
	$20,000 - $39,000	29	15%		63	15%
	$40,000 - $59,000	31	16%		67	16%
	$60,000 - $79,000	23	12%		68	16%
	$80,000 or higher	62	32%		120	28%
	I prefer not to say	32	16%		59	14%

Note. Some demographic information was not collected for the hierarchy validation survey in order to reduce survey length.

Trajectory Mapping

Trajectory Mapping (TM; [1]) is a non-metric scaling technique developed to elicit similarity relationships between categories. TM assumes that objects (e.g., AEs) are represented as discrete categories that can, but need not, be ranked relative to one another. This partially ranked approach allows the construction of equivalence classes – groups of outcomes that even if not directly comparable to one another, can all be compared relative to a common reference point. For example, TM allows patients to say, that while pneumonia and cataracts may not be directly comparable, they are both worse than heartburn.

We constructed AE hierarchies from TM data as follows: We first asked presented patients with a randomly selected pair of AEs (X and Y).

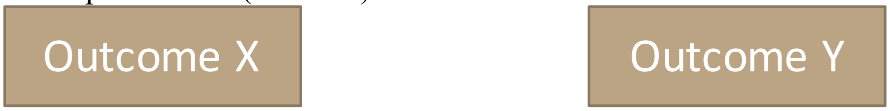

Given this pair, subjects were asked which outcome is worse, establishing a ranking. For example, when comparing severe fatigue to mild injection site skin reaction, a subject might indicate that severe fatigue is worse.

How different do you think these two side effects are?

Completely the same ○ ○ ○ ○ ○ ◉ ○ Completely different

Pair [1] of 11

Severe fatigue and/or insomnia	Mild injection site skin reaction or rash
(Severe or frequent enough to affect quality of life and/or limit activities. Frequency varies, but usually happens at least 2 to 3 days a week - regardless of the amount of activity or stress going on.)	*(Mild and tends to resolve with continued use of RA medication.)*

Which of these two side effects is **WORSE?** Again, please think in terms of what matters to you, personally.

- ○ Severe fatigue and/or insomnia
- ○ Mild injection site skin reaction or rash
- ○ I can't say which one is worse

Next, subjects were asked to identify the specify feature underlying their choice. For example, when comparing severe fatigue to mild injection site skin reaction, a subject might indicate that severe fatigue is worse because they interfere with daily activities.

In what way is **Severe fatigue and/or insomnia** worse than **Mild injection site skin reaction or rash** to you?

! You can see more information about the side effects above by clicking or hovering over them.

Please pick the **ONE most important** reason to you, personally.

- ○ If it affects your internal organs
- ○ How much it affects eating/appetite
- ○ How much it affects ability to concentrate
- ○ How much you notice the side effect
- ○ If it involves hospital time
- ○ Whether you need to continue monitoring it
- ○ How difficult it is to treat
- ○ How difficult lifestyle changes are (diet, exercise, etc.)
- ○ How long it continues to be a problem
- ○ How much it affects ability to do activities
- ○ If over-the-counter medication is needed
- ○ How often it occurs
- ○ Whether it could be life-threatening
- ○ If prescription medication is needed
- ○ How much it affects overall quality of life
- ○ If surgery is needed
- ○ How painful it is
- ○ Whether it affects your appearance
- ○ How long it lasts each time it happens
- ○ How stressful it is to experience
- ○ Something else (Please specify)

Having identified this feature, subjects are next asked to extrapolate outcomes using this same feature.

For the next few screens, you will be asked more questions about side effects.

It is very important that the side effects you pick are different (either better or worse) IN THIS WAY: How much it affects ability to do activities

Please confirm you have read and understand this instruction.

- ○ Yes, I understand I need to select side effects that are either better or worse **IN THIS WAY: How much it affects ability to do activities**

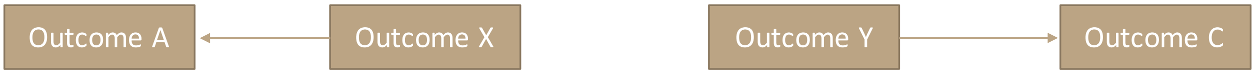

For example, subjects might be asked to select an AE (Outcome A, e.g. reversible blood test abnormalities) that interferes less with daily activities than mild injection site skin reaction or rash (Outcome X).

Choose **ONE** side effect from the list that is **NOT AS BAD** as **Mild injection site skin reaction or rash in the same way** as you just **selected**: **How much it affects ability to do activities**

! You can see more information about each side effect by clicking or hovering over it.

- ○ Medication-controlled high blood pressure, sugar, or cholesterol
- ○ Change in appearance (hair thinning or loss, or weight gain or loss)
- ○ Mild infection treated at home
- ○ Manageable depression and/or anxiety
- ○ Curable non-melanoma skin cancer
- ○ Manageable mouth ulcers
- ○ Manageable brain fog and/or lightheadedness
- ○ Severe brain fog and/or lightheadedness
- ○ Severe headaches
- ○ Serious infection treated in the hospital
- ○ Cataracts
- ○ Diet-controlled high blood pressure, sugar, or cholesterol
- ◉ Reversible blood test abnormalities
- ○ Shingles
- ○ Manageable stomach pain and/or diarrhea
- ○ Manageable headaches
- ○ Manageable nausea and/or vomiting
- ○ Manageable fatigue and/or insomnia
- ○ I can't find a side effect in this list

Is Reversible blood test abnormalities **NOT AS BAD** as Mild injection site skin reaction or rash in this way: **How much it affects ability to do activities**?

- ◉ Yes; it is not as bad **in this way**
- ○ No; it is not as bad in **some other way**

and an AE which interferes more (Outcome C, e.g., serious infection treated in the hospital) with daily activity than severe fatigue and/or insomnia (Outcome Y).

Now pick **ONE** side effect from the list that is **EVEN WORSE** than **Severe fatigue and/or insomnia in the same way: How much it affects ability to do activities**

It does not have to be the absolute worst side effect from the list, but it should be **EVEN WORSE** than **Severe fatigue and/or insomnia**.

! You can see more information about each side effect by clicking or hovering over it.

- ○ Medication-controlled high blood pressure, sugar, or cholesterol
- ○ Change in appearance (hair thinning or loss, or weight gain or loss)
- ○ Mild infection treated at home
- ○ Manageable depression and/or anxiety
- ○ Curable non-melanoma skin cancer
- ○ Manageable mouth ulcers
- ○ Manageable brain fog and/or lightheadedness
- ○ Severe brain fog and/or lightheadedness
- ○ Severe headaches
- ◉ Serious infection treated in the hospital
- ○ Cataracts
- ○ Diet-controlled high blood pressure, sugar, or cholesterol
- ○ Shingles
- ○ Manageable stomach pain and/or diarrhea
- ○ Manageable headaches
- ○ Manageable nausea and/or vomiting
- ○ Manageable fatigue and/or insomnia
- ○ I can't find a side effect in this list

Is Serious infection treated in the hospital **EVEN WORSE** than Severe fatigue and/or insomnia in this way: **How much it affects ability to do activities**?

- ◉ Yes; it is even worse **in this way**
- ○ No; it is not even worse **in some other way**

Finally, subjects were asked to select an AE that is intermediate between outcomes A and B in terms of this feature.

Lastly, pick **ONE** side effect from the list that **falls between Mild injection site skin reaction or rash** and **Severe fatigue and/or insomnia**. In other words, it should be **WORSE** than **Mild injection site skin reaction or rash**, but **NOT AS BAD** as **Severe fatigue and/or insomnia**.

Again, think about the way the side effects differ, which you selected earlier: How much it affects ability to do activities

! You can see more information about each side effect by clicking or hovering over it.

- ○ Medication-controlled high blood pressure, sugar, or cholesterol
- ○ Change in appearance (hair thinning or loss, or weight gain or loss)
- ○ Mild infection treated at home
- ○ Manageable depression and/or anxiety
- ○ Curable non-melanoma skin cancer
- ○ Manageable mouth ulcers
- ○ Manageable brain fog and/or lightheadedness
- ○ Severe brain fog and/or lightheadedness
- ○ Severe headaches
- ○ Cataracts
- ○ Diet-controlled high blood pressure, sugar, or cholesterol
- ○ Shingles
- ○ Manageable stomach pain and/or diarrhea
- ◉ Manageable headaches
- ○ Manageable nausea and/or vomiting
- ○ Manageable fatigue and/or insomnia
- ○ I can't find a side effect in this list

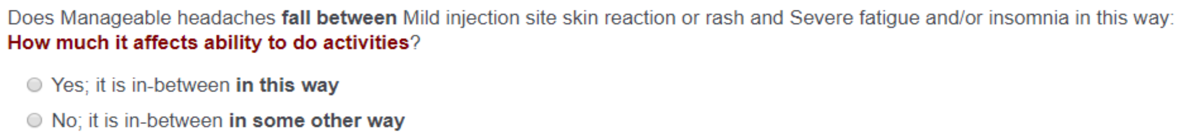

For example, manageable headaches (Outcome B) might interfere more with daily activities than mild injection site skin reaction or rash, but less than severe fatigue and/or insomnia.

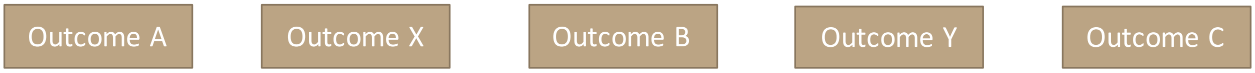

The result is an ordered quintuple A > X > B > Y > C. Finally, these quintuples are divided into triples, A > X > B, X > B > Y, B > Y > C, which are used as the unit of analysis to construct the AE hierarchies described below.

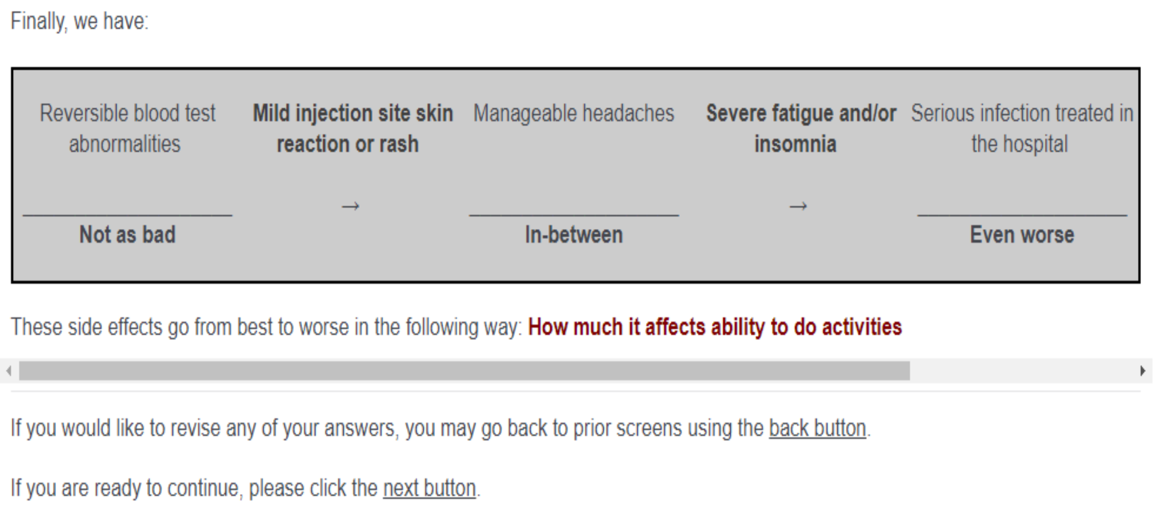

Power Analysis for Trajectory Mapping

The Frequency of Triples Follows a Poisson Distribution

Assuming a fixed number, n, of AEs, $n(n-1)/2$ pairs (A, B) must be compared in order to execute the TM procedure. Per the TM procedure, each such pair will be used to generate a quintuplet (X, A, Y, B, Z), which is then further decomposed into three triplets (X, A, Y), (A, Y, B), and (Y, B, Z). We wish to determine which triples in the TM procedure occur significantly more often than would be expected due to chance.

Assuming that each triple is equally likely to appear, and that order of the triple matters, there are $\frac{n!}{(n-3)!}$ possible triples. If these triples are selected at random from a uniform distribution (i.e., each are equally likely) without replacement, then the baseline probability that any given triple will appear at least k times follows a Binomial distribution:

$$P(k) = \left(\frac{(S*N)!}{k!(S*N-k)!} q^k (1-q)^{S*N-k}\right), \quad \text{(S1)}$$

where $q = \frac{1}{T}$, where S is the total number of subjects recruited, N is the number of triples generated by each subject (such that S*N is the total number of triples generated), and T is the total number of possible triples.

Power Analysis for 20 AEs

Assuming 20 AEs, there are 6,840 possible triples. Given N and T, we want to select a value of S that would allow us to determine if a given triple is at least 20 times more likely to appear in our data than we would expect due to chance (i.e., the probability that we observe that triple under the alternate hypothesis = 20/6840 = 0.3%). Using the G*Power software package (Faul et al., 2007; 2009), and assuming a false positive rate of □ = 0.05/6840 (using the Bonferroni correction for each triple to control for multiple comparisons) and a false negative rate of □=0.05, we calculated that a total sample size of 3,855 triples was required. Per this rubric, each triple that was seen 7 times or more would be considered statistically significant.

Some triples are not viable because subjects may indicate that there is no extrapolant or interpolant. Therefore, in order to determine the total number of pairs of AEs to show each subjects (from which they might generate quintuples, and therefore triples), we write a computer program in Python to calculate the average number of triples a given subject might generate given all 190 triples. The program generated simulated subjects, each of whom received a randomly-selected pair of 20 AEs, A-T. Next, simulated subjects randomly selected three AEs from this list of 20 options including the remaining 18 AEs, an option, X, indicating that that there is no extrapolant/interpolant available, and an option, 0, indicating that there is an extrapolant/interpolant available, but that it's not in the list, A-T. The results of this simulation indicated that a subject would generate an average of N=52.1 triples assuming random selection and given 20 pairs of AEs selected at random. Thus, our analysis indicated that we needed to recruit S = 74 = 3855/52.1 subjects, each of whom would generated quintuples from 20 pairs. We inflated the sample size by roughly 25% (to 95 subjects) to account for missing data and elimination of respondents completing the survey too quickly.

Upon piloting the survey, it became apparent that subjects would fatigue before completing all 20 pairs. Therefore, we reduced the number of pairs required from each subject to 11. In order to maintain the same average number of quintuples (and, therefore, triples), our minimum recruitment goal was increased to S = 20 * 95 / 11 = 173 subjects.

We closed the survey after successful enrollment of 200 respondents.

Trajectory Mapping Survey Results

Demographics and Descriptive Statistics

Two hundred patients finished the survey yielding 200 complete responses. Of these, 5 subjects were eliminated due to completing the survey too quickly, leaving responses of 195 patients included in the final sample for further analysis. 173 (89%) of these patients were female and 22 (11%) of them were male. The average age (SD) was 52.38 (11.42). 181 (93%) patients were Caucasian and 106 (54%) were college graduates. 29 (15%) patients rated their overall health status as excellent/very good and their average (SD) rating over past weekend was 6.04 (2.63) on an 11-point scale (0-very well, 10-very poor).

Frequency of Each Triple in TM Data

Our analysis yielded 2,808 unique triples (5,195 triples in total). Assuming $\alpha = 0.05$ and controlling for multiple comparisons using the Holm-Bonferroni procedure, we retained all ordered triples that were expressed at least 6 times (in practice, the Bonferroni and Holm-

Bonferroni criteria generated the same results). These triples, and their relative frequencies, are listed below:

Table S2: Frequency of each triple in the TM data.

Triple
Frequency = 13
MJA; TPX
Frequency = 12
MAN; NBP
Frequency = 11
MJN; ABO; AOP; DBP
Frequency = 10
CDB; LDB
Frequency = 9
IFD; ABP; NQP; RMN; MNA; KOB
Frequency = 8
KBP; MAJ; RTP; AOB; AGF; JAO; MED
Frequency = 7
MTI; MLA; FBD; AFP; MNE; MAC; NHP; GFP; AFB; CFP; EFD; MCA; MNJ; FDB; EFP; GDB; NOP; JNQ; QPX; EFB; MKN; MIG; KFP
Frequency = 6
MRT; CDP; CBD; EST; MNK; AJO; QOP; FBP; MJE; FDP; CFB; GCD; MRB; AKN; NCK
GEF; MEC; ALP; MGF; JKB; MJK; AMN; MKA; TQP; CKB; KNP; MRA; KOP; MRN; LAO; ADP; LKD; QHP; BOP; EDB; BPO; ADB

Note. AE IDs are as defined in Table 1. X = "no extrapolant/interpolant available", typically indicating that the adjacent AE is at the top or bottom of that subject's hierarchy.

Constructing Hierarchies from Triples

81 triples were retained because they occurred 6 times or more. Each dyad was then tallied to construct the adjacency matrix represented below (AEs have been grouped to reflect underlying structure). For example, the triple A, B, C would be represented as edges between A and B, and B and C.

	R	M	L	C	A	N	J	K	O	T	I	G	E	F	D	B	Q	H	S	P
R	1	1			1	1				1						1				
M	1	1	1	1	1	1	1	1			1	1	1							
L			1		1			1							1					1
C				1	1			1						1	1	1				
A		1	1		1	1	1	1	1			1		1	1	1				
N				1	1	1	1	1	1							1	1			1
J					1	1	1	1	1				1							
K					1	1		1	1					1	1	1				
O									1							1				1

T							1	1						1			1
I								1	1		1						
G	1								1	1	1	1					
E	1									1	1	1				1	
F											1	1	1				1
D												1	1				1
B						1						1	1				1
Q						1								1	1		1
H															1		1
S							1									1	
P						1											1

Figure S1: Adjacency matrix representing network edges between AEs. Letters represent AEs in Table 1.

This adjacency matrix suggests a trajectory map with an overall hierarchical structure. The figure below shows this structure with edges going opposite the direction of the structure shown (edges respecting the structure are not shown for readability).

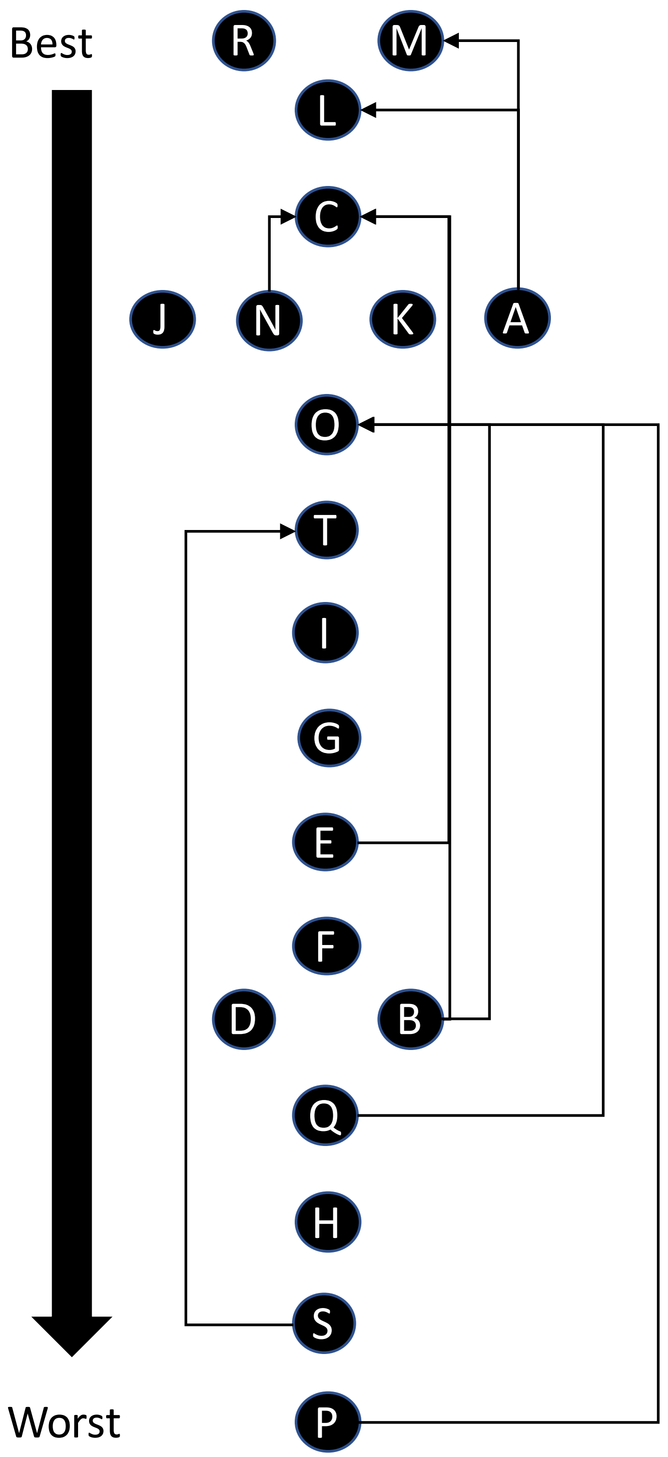

Figure S2: Hierarchical representation of adjancency matrix in Figure S1. Letters represent AEs in Table 1.

Methods for Generating Hierarchies

Given trajectory mapping data, we used several different techniques to generate candidate hierarchies. Specifically, the trajectory map was treated as a directed one-mode network where

nodes represented AEs and edges represented preference relationships. We used 5 different network analysis methods to extract structural properties of the triple data:

Block Modeling. We used the CONCOR algorithm [2] to group AEs into clusters that had similar roles in the network (e.g., if all AEs in one cluster were preferred to AEs in a second cluster). The output of the CONCOR algorithm, implemented in UCINet [3], is illustrated below.

```
                     1 1 1 1   1 1 2     1     1 1         1
       1 3   7 9 5   1 2 3 0   4 8 0   2 6   8 5 7   4 6   9
       A C   G I E   K L M J   N R T   B P   H O Q   D F   S
     ---------------------------------------------------------
 1 A | 1 1 | 1     | 1 1 1 2 | 1     | 2   |   4   | 2 2 |   |
 3 C | 1 1 |       | 2       |       | 1   |       | 3 2 |   |
     ---------------------------------------------------------
 7 G |   1 | 1   1 |         |       |     |       | 1 3 |   |
 9 I |     | 1 1   |         |       |     |       |   1 |   |
 5 E |   1 |     1 |         |       |     |       | 2 4 | 1 |
     ---------------------------------------------------------
11 K | 1   |       | 1       | 3     | 3   |   2   | 1 1 |   |
12 L | 2   |       | 1 1     |       |   1 |       | 1   |   |
13 M | 3 1 | 1 1 2 | 2 1 1 4 | 6 4 1 |     |       |     |   |
10 J | 2   |     1 | 2     1 | 2     |     |   1   |     |   |
     ---------------------------------------------------------
14 N | 1 1 |     1 | 1     1 | 1     | 1 1 | 1 1 2 |     |   |
18 R | 1   |       |     1   | 1 1 2 | 1   |       |     |   |
20 T |     |   1   |         |     1 |   1 |     1 |     |   |
     ---------------------------------------------------------
 2 B |     |       |         |       | 1 6 |   2   | 2   |   |
16 P |     |       |         |       |   1 |   1   |     |   |
     ---------------------------------------------------------
 8 H |     |       |         |       |   2 | 1     |     |   |
15 O |     |       |         |       | 2 5 |   1   |     |   |
17 Q |     |       |         |       |   2 | 1 1 1 |     |   |
     ---------------------------------------------------------
 4 D |     |       |         |       | 7 3 |       | 1   |   |
 6 F |     |       |         |       | 5 5 |       | 4 1 |   |
     ---------------------------------------------------------
19 S |     |       |         |     1 |     |       |     | 1 |
     ---------------------------------------------------------
```

Figure S3: Output of the CONCOR algorithm when applied to the network in Table S1, with each edge weighted by the number of times it occurs in the 81 quintuples retained.

Several hierarchies may be generated from these data, depending on selection of a cutoff value for the number of edges within a box. For example, one resulting hierarchy may be approximated as follows (edges respecting the structure are not shown for readability):

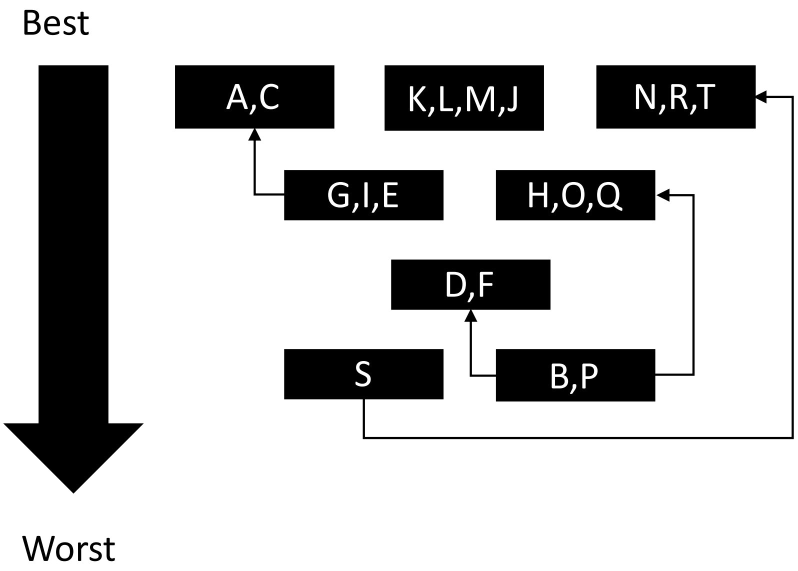

Figure S4: One example of a hierarchy generated from CONCOR data output shown in Figure S3.

Network partitioning. We used the Newman-Girvan algorithm[4] – a commonly-used "community finding" algorithm – to detect clusters of AEs that were likely to be connected to one another but loosely connected otherwise. This approach did not yield meaningful hierarchies.

Network Motifs. Network motifs [5] are defined as "patterns of interactions that occur at significantly higher rates in an actual network than in randomized networks". Among these motifs are several patterns indicative of hierarchy, such as a set of three nodes connected in a chain. We tested for significant 3-node and 4-node motifs using the FANMOD[6] software package. The significant 3- and 4-node motifs detected by FANMOD are illustrated in the table below. We used these motifs to identify discrete levels in our trajectory map, with the motifs themselves indicating the existence of different hierarchy levels.

Table S3: Listing of 3- and 4-node network motifs that occurred more often than would be expected due to chance. These motifs were extracted from the network shown in Table S1.

Motif	Observed Frequency %	Expected Frequency %	Standard Deviation	Z-Score
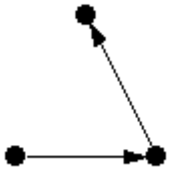	24.31	20.13	0.01	3.51***
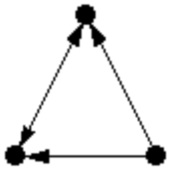	3.31	1.51	0.01	3.34***

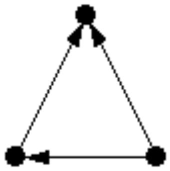	13.26	9.77	0.01	3.23^{**}
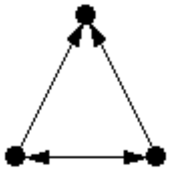	5.25	3.22	0.01	2.88^{**}
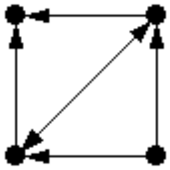	0.44	0.07	0.001	4.86^{***}
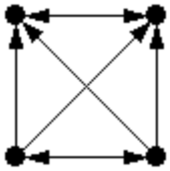	0.31	0.03	0.0005	5.89^{***}

The Shortest Path Algorithm. We observed that most subjects agreed that the adverse event "M- Mild injection site skin reaction or rash" was often most preferred, and that the adverse event "P- Serious infection treated in the hospital" was often least preferred. Thus, we calculated the shortest distance between each node and those 2 target nodes. Nodes with a minimum distance of n hops from node M were categorized into the same equivalence class, yielding one partially-ordered hierarchy. We repeated this procedure with node P as the origin node to generate a second hierarchy.

Flow Hierarchy. Luo and Magee [7] proposed a metric to measure the layers of nodes in a given information-flow network: "two nodes were in the same level, n, if they are reachable from a node in level n-1 or if they are in a cycle". We used this method to generate additional candidate hierarchies.

Overall, we created 11 hierarchies based on these different network analysis algorithms. Although some degree of judgment is required for each of these methods (e.g., in parameter selection), all of our generated hierarchies show convergent validity, *Spearman* $\rho > 0.89$, $p < 0.001$, for each pair of hierarchies.

Generating Data to Evaluate Hierarchies.

Each quintuple contains several pairs that may be used to evaluate the goodness-of-fit of each proposed hierarchy. For example, given the quintuple A > B > C > D > E, we may infer the following pairs:

1. A > B
2. A > C
3. A > D
4. A > E

5. B > C
6. B > D
7. B > E
8. C > D
9. C > E

Where "A>B" implies that A is preferred to B. Table S3, below, shows the number of subjects expressing a preference for each side effect

Table S4: Number of ratings derived from quintuples for each pairwise comparison

0	1	# Prefer 0	# Prefer 1	Total
A	B	147	0	147
A	C	71	58	129
A	D	143	10	153
A	E	64	40	104
A	F	117	10	127
A	G	81	15	96
A	H	45	9	54
A	I	55	28	83
A	J	55	52	107
A	K	79	31	110
A	L	81	38	119
A	M	37	162	199
A	N	83	57	140
A	O	123	4	127
A	P	202	1	203
A	Q	53	21	74
A	R	37	59	96
A	S	43	33	76
A	T	58	31	89
B	C	7	104	111
B	D	45	111	156
B	E	6	90	96
B	F	35	79	114
B	G	9	72	81
B	H	22	34	56
B	I	12	79	91
B	J	6	100	106
B	K	7	102	109

B	L	6	87	93
B	M	1	208	209
B	N	7	135	142
B	O	73	68	141
B	P	125	33	158
B	Q	18	40	58
B	R	5	103	108
B	S	5	69	74
B	T	14	70	84
C	D	116	6	122
C	E	36	46	82
C	F	92	9	101
C	G	47	25	72
C	H	43	10	53
C	I	34	28	62
C	J	31	47	78
C	K	62	32	94
C	L	53	41	94
C	M	27	129	156
C	N	54	64	118
C	O	68	7	75
C	P	152	6	158
C	Q	52	23	75
C	R	39	54	93
C	S	32	35	67
C	T	41	17	58
D	E	8	112	120
D	F	50	80	130
D	G	15	76	91
D	H	17	38	55
D	I	12	58	70
D	J	5	98	103
D	K	10	87	97
D	L	14	84	98
D	M	5	178	183
D	N	12	108	120
D	O	38	34	72
D	P	104	17	121
D	Q	12	53	65
D	R	9	88	97

D	S	13	68	81
D	T	3	44	47
E	F	89	4	93
E	G	30	28	58
E	H	22	7	29
E	I	24	21	45
E	J	26	42	68
E	K	44	25	69
E	L	49	21	70
E	M	12	109	121
E	N	28	49	77
E	O	56	3	59
E	P	98	1	99
E	Q	28	11	39
E	R	17	39	56
E	S	28	26	54
E	T	33	18	51
F	G	14	77	91
F	H	20	25	45
F	I	12	45	57
F	J	4	68	72
F	K	10	68	78
F	L	8	61	69
F	M	1	155	156
F	N	3	106	109
F	O	35	25	60
F	P	117	16	133
F	Q	15	37	52
F	R	5	75	80
F	S	6	45	51
F	T	14	48	62
G	H	25	13	38
G	I	24	22	46
G	J	13	29	42
G	K	25	40	65
G	L	23	31	54
G	M	8	100	108
G	N	16	53	69
G	O	57	3	60
G	P	101	6	107

G	Q	27	16	43
G	R	15	39	54
G	S	17	33	50
G	T	25	24	49
H	I	14	23	37
H	J	9	43	52
H	K	18	27	45
H	L	14	20	34
H	M	7	91	98
H	N	9	71	80
H	O	39	16	55
H	P	101	21	122
H	Q	33	46	79
H	R	9	52	61
H	S	13	36	49
H	T	18	30	48
I	J	27	42	69
I	K	32	25	57
I	L	21	23	44
I	M	18	120	138
I	N	43	37	80
I	O	64	15	79
I	P	109	6	115
I	Q	43	23	66
I	R	26	38	64
I	S	22	38	60
I	T	30	24	54
J	K	59	27	86
J	L	54	17	71
J	M	18	139	157
J	N	50	52	102
J	O	104	2	106
J	P	128	3	131
J	Q	53	16	69
J	R	31	37	68
J	S	24	25	49
J	T	39	19	58
K	L	45	43	88
K	M	7	127	134
K	N	37	61	98

K	O	76	6	82
K	P	139	2	141
K	Q	30	16	46
K	R	14	53	67
K	S	31	32	63
K	T	37	17	54
L	M	12	107	119
L	N	24	54	78
L	O	60	7	67
L	P	119	4	123
L	Q	32	17	49
L	R	17	41	58
L	S	22	21	43
L	T	37	21	58
M	N	168	34	202
M	O	169	3	172
M	P	302	0	302
M	Q	109	6	115
M	R	96	39	135
M	S	84	20	104
M	T	117	7	124
N	O	96	5	101
N	P	205	0	205
N	Q	77	19	96
N	R	45	49	94
N	S	44	28	72
N	T	60	26	86
O	P	108	41	149
O	Q	18	41	59
O	R	3	73	76
O	S	2	49	51
O	T	12	58	70
P	Q	18	125	143
P	R	7	159	166
P	S	4	103	107
P	T	10	148	158
Q	R	15	48	63
Q	S	17	36	53
Q	T	39	43	82
R	S	40	30	70

R	T	59	21	80
S	T	64	19	83

Note. Letter indices A-T represent AEs shown in Table 1.

Logistic Loss Method for Calculating Likelihood of Each Hierarchy

In order to select between different hierarchies, we must derive a measure of how well each hierarchy fits the pairwise comparison data. As in previous work,[8] we base our goodness-of-fit measure on a logistic loss function – a standard method for modeling preferences between binary decisions. Specifically, given a choice between two options, A and B, we model the probability that a given subject will choose option A is P(A,B) = $\frac{1}{1+e^{-kx(A,B)}}$, where x(A,B) is the distance between options A and B in the corresponding decision hierarchy. For example, if option A is on the 1st level of the hierarchy and option B is on the 3rd level, the x(A,B) = +2 (similarly, x(B,A) = -2). Here, a positive number indicates a preference over A for B and vice versa for a negative number. Furthermore, this model assumes that the log-odds of selecting the preferred option increases by a fixed amount for each additional level in the hierarchy. This fixed amount is given by the constant, k, which is estimated independently for each hierarchy to minimize the total l_2-norm-regularized loss, which is given by:

$$\mathcal{L} = -\sum_{A,B}\left[n_A * \ln\left(P(A,B)\right) + n_B * \ln\left(P(B,A)\right)\right] + k^2$$

where n_A is the number of subjects choosing option A over option B.

Next, for each hierarchy, we calculated its goodness-of-fit using two standard metrics, the Akaike Information Criterion (AIC), and Schwarz's Bayesian Information Criterion (BIC), where

$$AIC = 2(c + \mathcal{L})$$

and

$$BIC = c\ln(n) + 2\mathcal{L}$$

where c is the total number of degrees of freedom in each model and n is the total number of pairs compared. Finally, the total number of degrees of freedom, c, is given by a * (m-1) + 2 where each of the a=20 AEs must be uniquely assigned to one of m hierarchy levels.

Hierarchies generated

11 hierarchies were generated based on four network analysis techniques (Table S5; the Newman-Girvan algorithm had poor performance on the trajectory mapping data thus was excluded).

Table S5: 11 hierarchies generated from 4 network analysis methods.

Hierarchy	1*	2*	3*	4*	5	6	7	8	9	10	11
1	M	M	M	M	M	M	M	M	M	M	M
2	AJRNCES	ANJR	RAJNESCI	AJRNCES	SEAJR	AJR	AJR	AJRESCN	AKNJLRGIECS	AJR	-
3	TKIGL	GIECSLK	KLGHTQ	TKIGL	TIGKLCN	CNEKS	CNESK	TKLIG	QTH	NCES	RAJNESC

**4**	DFHQ	HQT	FDBO	QH	DFQH	TGIL	TGQH IL	QHDF BO	FD	TKLI GQ	KLIG H
**5**	BO	FD	P	DF	OB	DFQH	DF	P	OB	DFOH	TQ
**6**	P	BO	-	OB	P	OB	OB	-	P	B	FD
**7**	-	P	-	P	-	P	P	-	-	P	BO
**8**	-	-	-	-	-	-	-	-	-	-	P
**# of levels**	6	7	5	7	6	7	7	5	6	7	8
**AIC**	15426	**15335**	15451	15338	15739	15616	15558	15684	15641	15703	*15398*
**BIC**	16217	16281	**16087**	16284	16530	16563	16504	16320	16432	16650	16500

Note. All AEs are represented by the letter shown in Table 1. AIC = Akaike Information Criterion. BIC = Bayesian Information Criterion. The best-fitting hierarchy according to each criterion is in **bold.** Hierarchies 1-4 (indicated by asterisks) have the qualitatively lowest AIC and BIC values.

We also included two additional hierarchies to test for overfitting and underfitting. The first, intentionally overfit, hierarchy is "saturated", in which two AEs were put into different levels if there was any difference in their pairwise preference data (i.e., if the number of subjects preferring one AE exceeded the number of subjects preferring the second AE; Table S6).

Table S6: Saturated hierarchy

Hierarchy Level	AEs
**1**	M
**2**	R
**3**	A
**4**	J
**5**	N
**6**	E
**7**	S
**8**	C
**9**	KLIG
**10**	H
**11**	T
**12**	Q
**13**	F
**14**	D
**15**	B
**16**	O
**17**	P
**# of levels**	17
**AIC**	16195
**BIC**	18693

Note. All AEs are represented by the letter shown in Table 1. AIC = Akaike Information Criterion. BIC = Bayesian Information Criterion.

The second, intentionally underfit, hierarchy is "null" in which all adverse events were put into the same level, with values of AIC = 23966 and BIC = 23981.

AE Hierarchy Validation Survey

In this survey, subjects were asked to do 11 pairwise comparisons between groups of AEs. These 11 pairs were chosen because of their ability to adjudicate between our candidate set of hierarchies (see Table S7).

Table S7: 11 pairwise comparisons between groups of AEs in the AE hierarchy validation survey

Pair	*Group A*	*Group B*
1	AJNR	CES
2	GKL	CES
3	I	AJNR
4	I	CES
5	I	GKL
6	I	T
7	GKL	HQ
8	GKL	T
9	T	HQ
10	HQ	DF
11	DF	BO

For each pair of grouped AEs, subjects were instructed to rate their preferred option using a 7-point Likert scale

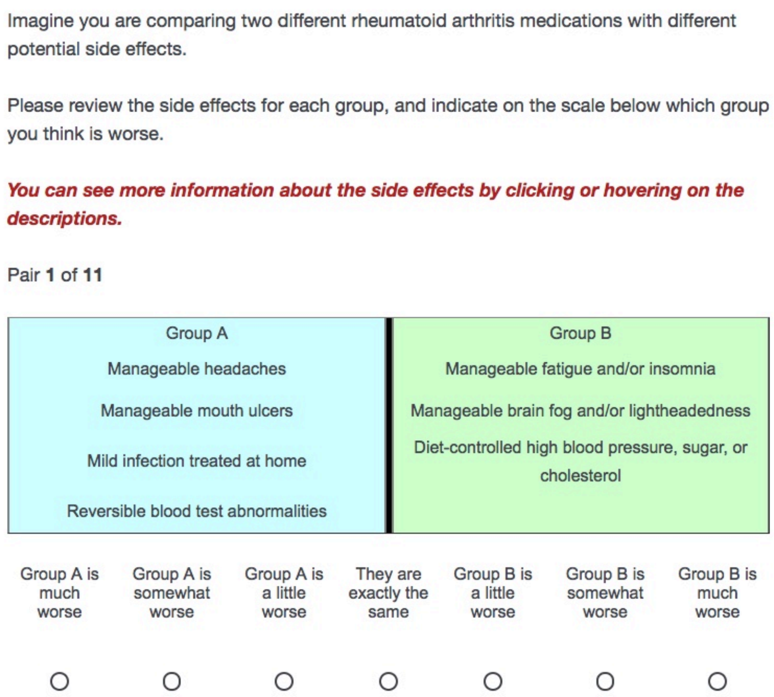
Imagine you are comparing two different rheumatoid arthritis medications with different potential side effects.

Please review the side effects for each group, and indicate on the scale below which group you think is worse.

You can see more information about the side effects by clicking or hovering on the descriptions.

Pair **1** of **11**

Group A	Group B
Manageable headaches	Manageable fatigue and/or insomnia
Manageable mouth ulcers	Manageable brain fog and/or lightheadedness
Mild infection treated at home	Diet-controlled high blood pressure, sugar, or cholesterol
Reversible blood test abnormalities	

Group A is much worse	Group A is somewhat worse	Group A is a little worse	They are exactly the same	Group B is a little worse	Group B is somewhat worse	Group B is much worse
○	○	○	○	○	○	○

Demographics and Descriptive Statistics.

One hundred and twenty-nine patients finished the survey yielding 129 complete responses. Of these, 2 were eliminated due to completing the survey too quickly, leaving responses of 127 (98%) patients in the final sample for further analysis. For all participants, 113 (89%) were female and 14 (11%) were male. The average age (SD) was 54.68 (10.89). To reduce survey length, we did not collect other demographic data.

Survey Results.

Numbers indicate subjects' preference in each pair of AE groups outlined in Table S7. Specifically, Group A is much worse (-3) Group A is somewhat worse (-2) Group A is a little worse (-1) They are the same (0) Group B is a little worse (+1) Group B is somewhat worse (+2) Group B is much worse (+3).

Table S8: Subject responses for each pairwise group comparison.

ID	*1*	*2*	*3*	*4*	*5*	*6*	*7*	*8*	*9*	*10*	*11*
1	-3	-1	2	0	0	0	3	0	3	-3	3
2	-3	3	-3	-3	-3	3	3	0	3	-3	3
3	-1	1	1	1	0	-2	3	-1	2	0	1
4	-2	-3	0	-2	0	0	0	-2	1	-2	3
5	1	1	1	-1	1	1	3	-1	3	-3	1
6	0	1	2	2	2	-2	3	-2	3	-3	2
7	2	-1	-3	0	1	1	0	-1	0	1	0
8	-3	-3	2	2	2	-2	2	-2	2	-1	0
9	0	0	-3	0	0	3	-3	-3	0	3	0
10	-3	-2	2	-2	-1	2	3	-2	3	-3	2

11	-1	1	2	2	1	1	2	-1	1	-1	2
12	2	2	1	1	-1	1	1	-1	3	2	2
13	0	3	-3	1	-3	3	-2	-1	3	-3	3
14	-1	-3	2	2	3	-2	0	-2	0	-3	2
15	3	2	3	3	2	0	2	1	1	0	-2
16	2	2	3	2	2	-1	3	-1	3	-3	3
17	2	0	-2	-2	-3	3	3	2	3	-2	2
18	0	2	3	3	2	-2	2	2	2	-2	2
19	-3	-3	-3	-3	3	0	3	-3	3	-3	3
20	-2	-2	2	-1	2	1	2	-2	2	-2	0
21	-2	-2	1	-1	1	-1	0	0	2	-2	1
22	-2	-3	2	-2	-1	2	3	-2	3	-3	3
23	1	-1	1	1	2	2	2	-2	3	-3	3
24	1	-2	1	-1	1	-2	3	-1	2	0	3
25	1	0	-1	1	0	-1	2	1	1	2	0
26	-2	-1	2	3	3	-3	1	-2	3	1	1
27	-2	1	1	1	-1	-2	3	2	3	-2	3
28	0	2	-2	-1	-3	0	3	3	0	-2	1
29	-2	-2	-2	-2	-1	2	-2	-1	1	2	2
30	0	0	-2	-2	-1	1	2	0	1	-1	0
31	1	1	1	1	-1	-2	2	0	1	1	-2
32	0	-1	-1	-1	0	0	0	-1	1	0	0
33	-2	-2	3	3	3	-2	3	0	3	0	3
34	3	-3	-3	2	2	2	-1	-2	1	2	0
35	0	-3	3	3	2	-2	3	0	2	0	3
36	-2	1	2	0	0	1	0	-2	1	0	1
37	2	-1	1	1	1	-1	-1	1	1	1	1
38	2	1	3	3	3	-3	3	0	3	-2	2
39	1	-2	0	-2	1	-1	0	-1	1	0	3
40	-1	-1	2	2	2	-2	1	0	0	3	-1
41	2	-1	-1	0	1	0	2	0	1	-2	2
42	-2	-3	-2	1	3	2	0	-2	2	0	2
43	2	-3	2	2	2	1	3	-3	3	-3	3
44	-1	-1	2	1	1	0	0	0	1	-1	0
45	3	3	-3	-2	-2	2	2	1	1	2	-1
46	-3	-3	-3	-1	-2	-1	3	-2	2	-2	3
47	3	-3	-2	-1	1	-3	2	1	2	0	1
48	-2	1	2	2	2	-2	2	-1	0	0	3
49	1	-1	1	1	1	-1	-1	1	1	2	1
50	0	0	0	0	0	0	2	1	0	-2	2

51	-1	-2	1	1	1	-1	0	-1	0	-1	2
52	1	1	2	2	1	-3	3	2	-1	0	3
53	-2	-1	1	-2	-2	2	2	0	2	-1	3
54	2	-1	-3	-1	3	3	-1	-1	0	3	-1
55	-3	-2	-2	-2	-2	2	3	-1	3	-3	2
56	-2	-2	1	1	1	1	3	-2	3	-2	2
57	-1	-2	1	1	1	-1	-1	-1	1	2	0
58	2	-1	-2	-1	0	1	2	2	0	-1	2
59	2	0	-3	-3	1	2	3	-2	3	-2	3
60	3	3	-1	2	2	1	2	0	1	3	0
61	-3	-3	3	2	3	-3	3	-1	3	-1	3
62	1	-3	-1	-1	1	1	-1	-3	2	0	0
63	-2	-3	1	0	3	1	-1	-3	1	2	3
64	-3	-3	2	2	2	0	0	-1	1	2	0
65	-2	-3	-2	-2	3	2	0	-3	3	-3	3
66	-3	-3	2	3	3	-3	3	-3	3	3	2
67	-3	-3	3	-1	-1	1	3	-1	1	-2	3
68	1	-2	-3	-2	-2	2	2	0	2	1	3
69	-3	-3	0	-1	3	2	2	-2	2	0	3
70	2	1	1	1	1	-2	-2	-2	-1	1	3
71	1	2	2	-3	3	3	3	-3	-3	3	-3
72	-1	-2	2	1	1	-1	1	0	1	-1	2
73	1	-2	-2	-2	-2	2	-1	-1	2	2	3
74	-3	-3	-2	-2	0	1	-1	-2	1	2	0
75	0	1	3	3	-3	3	3	3	3	0	0
76	-3	1	2	2	1	0	-1	-1	2	0	3
77	-3	1	1	1	1	-1	3	-2	2	1	2
78	2	1	-3	-3	-3	3	-3	3	-3	3	-3
79	-2	-1	-2	-2	-2	2	2	0	0	3	1
80	-1	-2	-1	-1	-1	1	3	2	2	2	2
81	0	-1	1	1	1	0	1	-1	1	2	0
82	2	2	1	2	1	-1	2	1	1	-2	1
83	2	-2	-2	-1	2	-1	2	-2	2	-2	2
84	1	-2	3	3	3	3	-3	-3	-3	1	-2
85	-3	-3	1	2	2	-3	3	-2	3	-3	2
86	0	-1	1	1	1	-1	1	-1	2	1	2
87	-2	-2	-1	-1	-1	-1	3	-1	3	-1	2
88	-3	-3	3	-3	3	3	3	-3	3	-3	3
89	-3	-3	-2	-3	0	1	2	-3	3	0	1
90	-2	-3	1	2	3	0	3	-2	3	0	3

91	2	2	0	0	0	-3	3	3	0	-3	2
92	1	2	-1	1	2	2	1	-2	1	0	3
93	2	-2	-3	-3	2	3	-2	-2	2	3	3
94	0	-3	1	1	3	1	-1	-1	2	2	-2
95	2	-2	-3	-3	2	2	3	-3	2	3	-3
96	2	2	-1	-1	-2	0	3	0	3	0	0
97	1	-1	2	2	3	-1	0	-1	2	-2	0
98	-1	-3	-1	-2	0	0	1	-1	1	-2	2
99	2	2	-2	2	-1	-1	3	2	3	-3	2
100	0	0	2	0	1	0	0	0	2	3	0
101	-1	-1	-2	-2	0	0	2	0	2	1	3
102	-2	-2	-1	-1	-2	1	3	-2	3	-2	3
103	-3	-3	2	-2	3	2	0	-3	3	-2	2
104	1	1	1	1	1	-3	3	-1	2	2	0
105	2	2	2	3	3	0	-2	-3	0	-2	0
106	1	1	-1	1	-1	-2	3	2	2	0	-1
107	-3	-3	0	-2	2	-1	1	-2	3	-3	3
108	1	-1	1	1	1	1	2	-1	2	2	2
109	-2	-1	2	0	1	1	3	-2	3	-1	2
110	-2	2	1	1	1	-2	2	0	0	-2	0
111	-2	-1	2	1	1	-1	1	0	3	-3	3
112	1	0	1	0	2	-2	-1	-1	1	1	2
113	-3	-3	-2	-2	0	-2	3	1	1	-1	2
114	0	-3	2	2	3	-2	0	-2	2	-3	0
115	-1	-2	1	-1	-1	-1	1	1	2	2	2
116	-2	-1	2	1	1	-1	2	2	2	-3	0
117	-2	-2	2	-2	2	2	3	-1	3	-3	2
118	-2	-3	1	2	3	0	-1	-1	1	1	3
119	-1	2	0	0	0	-1	3	1	2	-2	3
120	1	1	2	2	2	-1	2	-3	2	3	1
121	-2	-3	-2	-3	-3	3	3	-3	3	-3	3
122	2	-1	-1	-1	1	1	2	-1	2	3	0
123	-2	0	1	1	2	0	2	-1	2	0	2
124	2	2	1	1	1	-2	3	1	3	-3	2
125	2	0	2	2	3	-2	3	2	0	-2	3
126	-2	-2	-1	-3	1	3	3	-3	3	-3	3
127	-3	1	-2	-3	-3	-1	3	2	3	-3	3

Note. ID = Subject identifier. Numbers 1-11 indicate each of the pairs indexed in Table S7.

Evaluation of Candidate Hierarchies on AE Valiadtion Survey Data Results

We tested the fitness of the four proposed hierarchies on the Likert score data presented above by calculating the likelihood of the Likert score data given a Gaussian model derived from each candidate hierarchy. Specifically, our model predicted that the Likert score given two groups of AEs was given by $\mathcal{L}(d) = \frac{1}{s\sqrt{2\pi}} e^{-\frac{1}{2}\left(\frac{k*d}{s}\right)^2}$ where d is the distance between a pair of AE groups (0 or 1 for the groups tested), k is a proportionality constant, and s is the empirical standard deviation in subjects' responses. For example, if a given hierarchy posited that two groups of AEs were on the same hierarchy level, the mean Likert score was expected to be k*d=0, whereas if they were one level apart, the score would be k*d=k. For each hierarchy, the value of k was chosen to optimize the likelihood. Finally, values of AIC and BIC were calculated using these likelihood values. The results show that among all four proposed hierarchies, "hierarchy 1" (with six levels) has the best likelihood (results replicated across multiple methodologies. For example, we also dichotomized Likert scale responses and used the logistic likelihood model employed for the TM survey).

Table S8: Hierarchy evaluation results for the AE validation survey.

Hierarchy	1*	2*	3*	4*
**1**	M	M	M	M
**2**	AJRNCES	AJRN	AJRNESCI	AJRNCES
**3**	TKIGL	GIECSLK	KLGHTQ	TKIGL
**4**	DFHQ	HQT	FDBO	QH
**5**	BO	FD	P	DF
**6**	P	BO		BO
**7**		P		P
**8**				
**Levels**	6	7	5	7
**AIC(GAU)**	**5511**	5690	5723	5550
**AIC(LOG)**	1550	1640	**1530**	1628
**BIC(LOG)**	**1641**	1731	1721	1719

Note. AIC = Akaike Information Criterion. BIC = Bayesian Information Criterion. GAU = Gaussian likelihood. LOG = Logistic likelihood. The best model according to each criterion is in **bold**.

The final hierarchy was selected based on the minimal Gaussian AIC and logistic BIC value (and the second-lowest logistic AIC value), which indicates a good fit on the survey 2 data.

Clinical Interpretation

We further added three adverse events into this hierarchy based on observations of clinical data. Specifically, "no adverse events" was added as the new best level and two more serious adverse events were added as the worst two levels. Additionally, the AE, I: Change in appearance, was split into two AEs because the data in Table S7 indicated a bimodal distribution of preferences compared to the AEs in levels 3 and 4. The final hierarchy for adverse events is listed below.

AE Level	
**1**	No side effect
**2**	Mild injection site skin reaction or rash

**3**	Manageable headaches	Manageable mouth ulcers	Mild infection treated at home	Reversible blood test abnormalities	Manageable fatigue and/or insomnia	Manageable brain fog and/or light-headedness	Diet-controlled high blood pressure, sugar, or cholesterol	Mild change in appearance (hair thinning or loss, or weight gain or loss)
**4**	Manageable depression and/or anxiety	Manageable stomach pain and/or diarrhea	Manageable nausea and/or vomiting	Medication-controlled high blood pressure, sugar, or cholesterol	Moderate change in appearance (hair thinning or loss, or weight gain or loss)			
**5**	Cataracts	Curable non-melanoma skin cancer	Severe fatigue and/or insomnia	Severe brain fog and/or lightheadedness				
**6**	Severe headaches	Shingles						
**7**	Serious infection treated in the hospital							
**8**	Serious complication from which you fully recover (like a bowel perforation that requires emergency surgery)							
**9**	Serious complication that is not fully reversible (like a neurologic disease that needs ongoing treatment)							

Global Hierarchy Survey

We conducted a paired comparison survey to build a hierarchy that organizes the combinations of AEs and levels of benefit. An example question is displayed below.

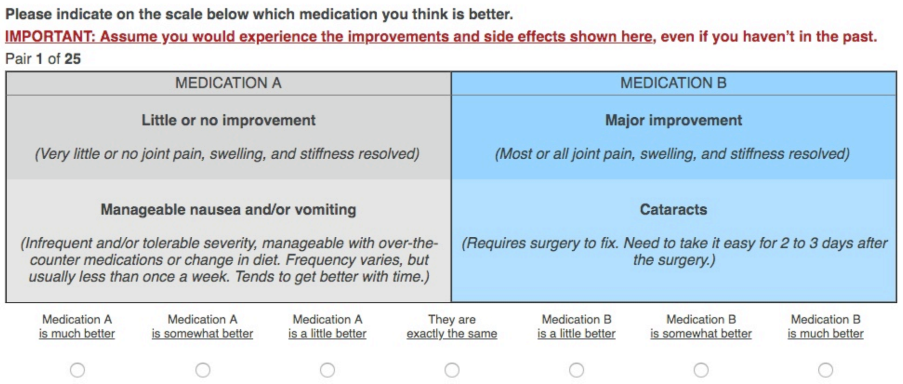

Demographics and Descriptive Statistics

Four hundred and fifty-three patients finished the survey yielding 453 complete responses. Of these, 27 were discarded due to completing the survey too quickly, leaving responses from 426 (94%) patients included in the final sample for further analysis. For all remaining patients, 389 (91%) were female and 37 (9%) were male. The average (SD) age was 53.78 (11.25). 395 (93%) of the final participants were Caucasian and 226 (53%) of them were college graduates. 56 (13%) participants rated their overall health status as excellent/very good and their average (SD) rating over past weekend was 4.89 (2.28) on an 11-point scale (0-very well, 10-very poor).

Results

Table S9 shows pairwise comparison results of the Global Hierarchy Survey (see Figure S5 for a graphical representation).

Table S1: T-test results comparing pairs of medications with varying levels of AE and benefit (ACR).

Medication 1		Medication 2							
AE	ACR	AE	ACR	N	Mean	SD	SE	t	p
1	2	2	1	115	2.46	1.19	0.11	22.10	<0.001*
1	2	3	1	91	1.41	1.84	0.19	7.28	<0.001*
1	2	4	1	96	1.01	1.90	0.19	5.21	<0.001*
1	2	5	1	104	0.02	1.97	0.19	0.10	0.921
1	2	6	1	93	-0.44	1.91	0.20	-2.22	0.029
1	2	7	1	97	-1.09	1.60	0.16	-6.72	<0.001*
1	2	8	1	98	-0.73	1.63	0.17	-4.45	<0.001*
1	2	9	1	96	-1.79	1.39	0.14	-12.62	<0.001*
1	3	2	1	83	-2.63	0.89	0.10	-26.79	<0.001*
1	3	2	2	111	2.11	1.13	0.11	19.64	<0.001*
1	3	3	1	125	1.98	1.36	0.12	16.29	<0.001*
1	3	3	2	98	1.31	1.34	0.14	9.63	<0.001*
1	3	4	1	100	1.58	1.36	0.14	11.64	<0.001*
1	3	4	2	97	0.91	1.76	0.18	5.09	<0.001*
1	3	5	1	96	0.88	1.65	0.17	5.20	<0.001*
1	3	5	2	97	0.31	1.54	0.16	1.97	0.051
1	3	6	1	99	0.80	1.54	0.15	5.16	<0.001*
1	3	6	2	95	0.03	1.47	0.15	0.21	0.835
1	3	7	1	90	0.21	1.74	0.18	1.15	0.252
1	3	7	2	91	-0.23	1.56	0.16	-1.41	0.163
1	3	8	1	94	0.40	1.74	0.18	2.26	0.026
1	3	8	2	92	-0.15	1.39	0.14	-1.05	0.297
1	3	9	1	89	-0.10	1.27	0.13	-0.75	0.455
1	3	9	2	97	-0.86	1.33	0.14	-6.33	<0.001*
2	2	3	1	108	1.79	1.43	0.14	12.95	<0.001*
2	2	4	1	89	1.43	1.66	0.18	8.12	<0.001*
2	2	5	1	98	0.14	1.80	0.18	0.78	0.435
2	2	6	1	95	-0.40	2.03	0.21	-1.92	0.058
2	2	7	1	96	-0.90	1.70	0.17	-5.16	<0.001*
2	2	8	1	91	-0.45	1.86	0.19	-2.31	0.023
2	2	9	1	88	-1.07	1.44	0.15	-6.97	<0.001*
2	3	3	1	91	-2.02	1.27	0.13	-15.15	<0.001*
2	3	3	2	116	1.28	1.42	0.13	9.65	<0.001*
2	3	4	1	112	1.58	1.67	0.16	10.02	<0.001*
2	3	4	2	99	1.22	1.51	0.15	8.06	<0.001*

2	3	5	1	95	0.94	1.66	0.17	5.52	<0.001*
2	3	5	2	94	0.30	1.41	0.14	2.06	0.043
2	3	6	1	104	0.49	1.66	0.16	3.01	0.003
2	3	6	2	104	-0.24	1.46	0.14	-1.67	0.097
2	3	7	1	95	-0.24	1.68	0.17	-1.40	0.164
2	3	7	2	98	-0.54	1.40	0.14	-3.82	<0.001*
2	3	8	1	96	0.40	1.57	0.16	2.48	0.015
2	3	8	2	89	-0.26	1.40	0.15	-1.74	0.086
2	3	9	1	94	-0.43	1.50	0.15	-2.75	0.007
2	3	9	2	103	-0.90	1.27	0.13	-7.20	<0.001*
3	2	4	1	113	1.27	1.73	0.16	7.79	<0.001*
3	2	5	1	97	0.19	1.85	0.19	0.99	0.326
3	2	6	1	88	-0.11	1.74	0.19	-0.61	0.541
3	2	7	1	88	-0.80	1.67	0.18	-4.47	<0.001*
3	2	8	1	102	-0.60	1.60	0.16	-3.78	<0.001*
3	2	9	1	87	-1.48	1.35	0.14	-10.28	<0.001*
3	3	4	1	100	-1.81	1.50	0.15	-12.10	<0.001*
3	3	4	2	114	0.97	1.36	0.13	7.65	<0.001*
3	3	5	1	108	1.00	1.57	0.15	6.62	<0.001*
3	3	5	2	97	0.49	1.41	0.14	3.46	0.001*
3	3	6	1	86	0.49	1.65	0.18	2.74	0.007
3	3	6	2	93	0.09	1.36	0.14	0.61	0.542
3	3	7	1	93	0.32	1.33	0.14	2.34	0.021
3	3	7	2	93	-0.49	1.32	0.14	-3.63	<0.001*
3	3	8	1	88	0.28	1.47	0.16	1.81	0.073
3	3	8	2	84	-0.10	1.34	0.15	-0.65	0.517
3	3	9	1	88	-0.33	1.33	0.14	-2.33	0.022
3	3	9	2	94	-0.88	1.07	0.11	-8.03	<0.001*
4	2	5	1	107	0.33	1.71	0.17	1.97	0.051
4	2	6	1	100	0.10	1.64	0.16	0.61	0.542
4	2	7	1	96	-0.59	1.63	0.17	-3.56	<0.001*
4	2	8	1	97	-0.06	1.71	0.17	-0.36	0.723
4	2	9	1	98	-0.90	1.40	0.14	-6.34	<0.001*
4	3	5	1	90	-0.86	1.50	0.16	-5.43	<0.001*
4	3	5	2	114	0.79	1.47	0.14	5.75	<0.001*
4	3	6	1	110	0.75	1.46	0.14	5.42	<0.001*
4	3	6	2	95	0.25	1.31	0.13	1.88	0.064
4	3	7	1	101	0.28	1.42	0.14	1.97	0.052
4	3	7	2	87	-0.30	1.26	0.13	-2.21	0.029
4	3	8	1	89	0.54	1.57	0.17	3.23	0.002

4	3	8	2	90	-0.12	1.36	0.14	-0.86	0.395
4	3	9	1	97	-0.21	1.25	0.13	-1.63	0.107
4	3	9	2	88	-0.53	1.14	0.12	-4.38	<0.001*
5	2	6	1	111	0.42	1.39	0.13	3.22	0.002
5	2	7	1	97	0.02	1.34	0.14	0.15	0.880
5	2	8	1	91	0.47	1.34	0.14	3.35	0.001
5	2	9	1	94	-0.89	1.15	0.12	-7.54	<0.001*
5	3	6	1	98	-0.96	1.40	0.14	-6.79	<0.001*
5	3	6	2	118	0.75	1.12	0.10	7.29	<0.001*
5	3	7	1	112	0.73	1.36	0.13	5.69	<0.001*
5	3	7	2	94	0.04	0.93	0.10	0.45	0.657
5	3	8	1	97	0.87	1.34	0.14	6.35	<0.001*
5	3	8	2	94	0.17	1.28	0.13	1.29	0.202
5	3	9	1	98	-0.24	1.29	0.13	-1.88	0.064
5	3	9	2	97	-0.33	0.99	0.10	-3.29	0.001
6	2	7	1	119	-0.08	1.47	0.14	-0.56	0.577
6	2	8	1	87	0.37	1.50	0.16	2.28	0.025
6	2	9	1	96	-0.28	1.32	0.13	-2.09	0.039
6	3	7	1	96	-0.72	1.48	0.15	-4.74	<0.001*
6	3	7	2	119	0.13	1.21	0.11	1.14	0.259
6	3	8	1	119	0.80	1.48	0.14	5.90	<0.001*
6	3	8	2	92	0.34	1.22	0.13	2.66	0.009
6	3	9	1	97	-0.19	1.11	0.11	-1.64	0.103
6	3	9	2	104	-0.31	1.19	0.12	-2.63	0.010
7	2	8	1	120	0.55	1.29	0.12	4.67	<0.001*
7	2	9	1	97	-0.20	1.05	0.11	-1.84	0.069
7	3	8	1	91	-0.92	1.21	0.13	-7.26	<0.001*
7	3	8	2	115	0.58	1.17	0.11	5.34	<0.001*
7	3	9	1	114	0.17	1.12	0.10	1.59	0.115
7	3	9	2	93	-0.06	1.01	0.10	-0.62	0.539
8	2	9	1	115	-0.37	1.15	0.11	-3.48	<0.001*
8	3	9	1	100	-0.18	0.99	0.10	-1.82	0.072
8	3	9	2	104	-0.14	1.14	0.11	-1.30	0.198

Note. *=significant after multiple comparisons at the 0.05 level after applying the Holm-Bonferroni procedure. AE = adverse event. ACR = American College of Rheumatology level of benefit.

	21	31	41	51	61	71	81	91	12	22	32	42	52	62	72	82	92	13	23	33	43	53	63	73	83	93
11	<	<	<	<	<	<	<	<	<	<	<	<	<	<	<	<	<	<	<	<	<	<	<	<	<	<
21	=	<	<	<	<	<	<	<	<	<	<	<	<	<	<	<	<	<	<	<	<	<	<	<	<	<
31		=	<	<	<	<	<	<	<	<	<	<	<	<	<	<	<	<	<	<	<	<	<	<	<	<
41			=	<	<	<	<	<	<	<	<	<	<	<	<	<	<	<	<	<	<	<	<	<	<	<
51				=	<	<	<	<	=	=	=	=	<	<	<	<	<	<	<	<	<	<	<	<	<	<
61					=	<	<	<	=	=	=	=	=	<	<	<	<	<	=	=	<	<	<	<	<	<
71						=	<	<	^	^	^	^	=	=	<	<	<	=	=	=	=	^	^	<	<	<
81							=	<	^	=	^	=	=	=	<	<	<	=	=	=	=	<	<	<	<	<
91								=	^	^	^	^	^	=	=	^	<	=	=	=	=	=	=	=	=	<
12									=	<	<	<	<	<	<	<	<	<	<	<	<	<	<	<	<	<
22										=	<	<	<	<	<	<	<	<	<	<	<	<	<	<	<	<
32											=	<	<	<	<	<	<	<	<	<	<	<	<	<	<	<
42												=	<	<	<	<	<	<	<	<	<	<	<	<	<	<
52													=	<	<	<	<	=	=	<	<	<	<	<	<	<
62														=	<	<	<	=	=	=	=	<	<	<	<	<
72															=	<	<	=	^	^	=	=	=	<	<	<
82																=	<	=	=	=	=	=	=	<	<	<
92																	=	^	^	^	^	=	=	=	=	<
13																		=	<	<	<	<	<	<	<	<
23																			=	<	<	<	<	<	<	<
33																				=	<	<	<	<	<	<
43																					=	<	<	<	<	<
53																						=	<	<	<	<
63																							=	<	<	<
73																								=	<	<
83																									=	<

Figure S5: Chart showing the aggregate results of the Step 3 Survey. Each AE-ACR profile is coded as follows: a profile has code XY, where X is the level in the AE hierarchy and Y is the level in the ACR hierarchy. For example, code 32 corresponds to the third-best AE level and the 2nd best ACR level. Comparisons in color were directly measured; all others were imputed because they are strictly dominated by other items in our hierarchy (e.g., 83 is expected to be uniformly preferred to 82, which is preferred to 72, etc.) < indicates the profile indexed by the row is preferred. ^ indicates the profile indexed by the column is preferred. = indicates no significant preference after controlling for multiple comparisons using the Holm-Bonferroni procedure.

These results indicate that ratings for AEs, when paired with benefits, cluster into four groups: No, mild or manageable AEs (Levels 1-4), Moderate AEs (Levels 5 and 6), Serious AEs (Levels 7 and 8), and Irreversible serious AEs (Level 9). This determination was made based on inspection of Figure S5, which indicates that, when comparing between levels of benefit, levels of AE that are in the same cluster behave the same way. For example, a medication with AE level 3 and ACR level 2 is worse than any medication with AE levels 1-4 (in the same cluster) but ACR level 1 (a better ACR level) , equivalent to medications with AE levels 5 and 6 (in a

worse cluster), but ACR level 1, and preferred to medications with AE levels 7-9, and ACR level 1.

Order Effect Validation Survey

We conducted a survey to test whether the order of in which medication profiles were displayed to subjects in the Global Hierarchy Survey may affect our results. For all pairs of profiles(combinations of benefits and adverse events) included in the Global Hierarchy Survey , we randomly selected specific AEs from each profile. Then, we randomly selected which the order in which these profiles would be displayed to each subject in this survey. Each pair appeared in both orders (i.e. profile A appeared on either left side or right side in a question.)

For example, some subjects saw a medication with Major improvement and Shingles on the left side and a medication with little or no improvement and mild infection treated at home on the right side. Some subjects will saw two outcomes in a reversed order.

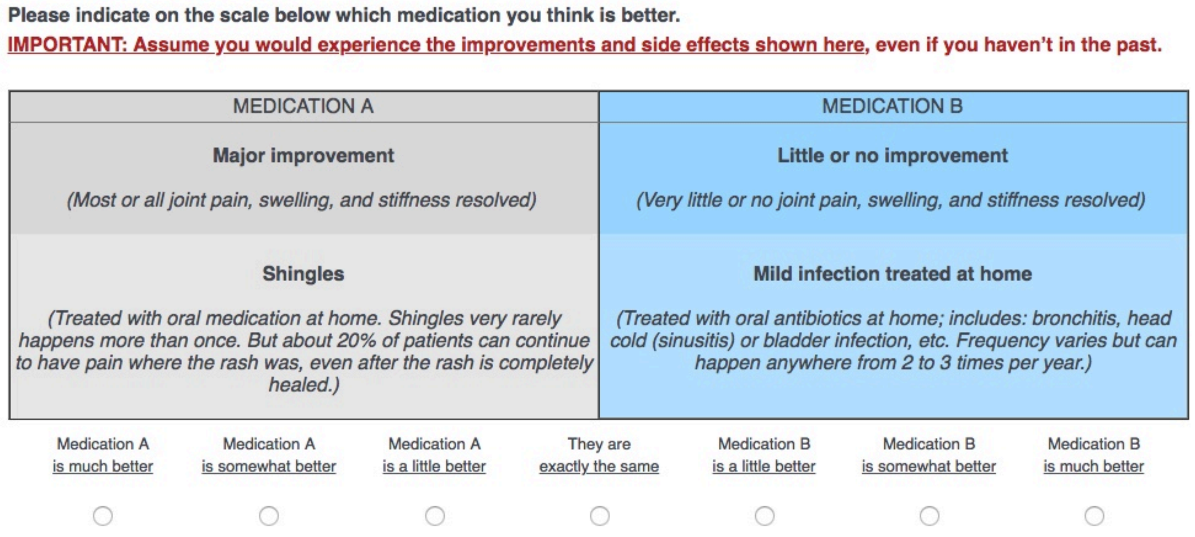
Please indicate on the scale below which medication you think is better.

IMPORTANT: Assume you would experience the improvements and side effects shown here, even if you haven't in the past.

MEDICATION A	MEDICATION B
**Major improvement** (Most or all joint pain, swelling, and stiffness resolved)	**Little or no improvement** (Very little or no joint pain, swelling, and stiffness resolved)
**Shingles** (Treated with oral medication at home. Shingles very rarely happens more than once. But about 20% of patients can continue to have pain where the rash was, even after the rash is completely healed.)	**Mild infection treated at home** (Treated with oral antibiotics at home; includes: bronchitis, head cold (sinusitis) or bladder infection, etc. Frequency varies but can happen anywhere from 2 to 3 times per year.)

Medication A is much better	Medication A is somewhat better	Medication A is a little better	They are exactly the same	Medication B is a little better	Medication B is somewhat better	Medication B is much better
○	○	○	○	○	○	○

Demographics and Descriptive Statistics

Two hundred and nine patients finished the survey yielding 209 complete responses. Of these, 7 of were discarded due to completing the survey too quickly, leaving responses from 202 (97%) patients included in the final sample for further analysis. For all remaining patients, 186 (92%) were female and 16 (8%) were male. The average (SD) age was 57.74 (11.18). 185 (92%) of the final participants were Caucasian and 103 (51%) were college graduates. 23 (11%) participants rated their overall health status as excellent/very good and their average (SD) rating over the past weekend was 5.14 (2.35) on a 11-point scale (0-very well, 10-very poor).

Results

Two sample t tests were used to test if there is a statistically significant difference between the order in which the two medications were displayed. None of the 22 pairs included in this survey rejected the null hypothesis which indicates that we did not detect an order effect.

References

1. Richards W, Koenderink JJ. Trajectory mapping: a new nonmetric scaling technique. Perception. 1995;24: 1315–1331.

2. Breiger RL, Boorman SA, Arabie P. An algorithm for clustering relational data with applications to social network analysis and comparison with multidimensional scaling. J Math Psychol. 1975;12: 328–383.

3. Borgatti SP, Everett MG, Freeman LC. Ucinet. Encycl Soc Netw Anal Min. 2014; 2261–2267.

4. Newman ME, Girvan M. Finding and evaluating community structure in networks. Phys Rev E. 2004;69: 026113.

5. Milo R, Shen-Orr S, Itzkovitz S, Kashtan N, Chklovskii D, Alon U. Network motifs: simple building blocks of complex networks. Science. 2002;298: 824–827.

6. Wernicke S, Rasche F. FANMOD: a tool for fast network motif detection. Bioinformatics. 2006;22: 1152–1153.

7. Luo J, Magee CL. Detecting evolving patterns of self-organizing networks by flow hierarchy measurement. Complexity. 2011;16: 53–61. doi:10.1002/cplx.20368

8. Broniatowski DA. Do design decisions depend on "dictators"? Res Eng Des. 2018;29: 67–85. doi:10.1007/s00163-017-0259-2
